# Host-directed therapeutics against antimicrobial-resistant bacterial infections: emerging roles of multi-omics and systems biology

**DOI:** 10.3389/fphar.2026.1916875

**Published:** 2026-09-23

**Authors:** Sarmistha Saha, Nadezhda Sachivkina, Oksana Gizinger, Nataliia Golikova, Asiyat Abdullaeva, Maria Grushko, Maria Molchanova

**Affiliations:** 1 Department of Biotechnology, Institute of Applied Sciences and Humanities, GLA University, Mathura, Uttar Pradesh, India; 2 Department of Microbiology V.S. Kiktenko, Institute of Medicine, Peoples’ Friendship University of Russia Named After Patrice Lumumba (RUDN University), Moscow, Russia; 3 Department of Medical Sociology, Health Economics, and Health Insurance, First Moscow State Medical University (Sechenov University), Moscow, Russia; 4 Department of Healthcare Organization, Moscow Medical University Reaviz, Moscow, Russia; 5 Department of Veterinary Sanitary Expertise and Biological Safety, Russian Biotechnology University (BIOTECH University), Moscow, Russia; 6 Department of Foreign Languages, Institute of Medicine, Peoples’ Friendship University of Russia Named After Patrice Lumumba (RUDN University), Moscow, Russia

**Keywords:** antibiotic resistance, host-directed therapies, host-pathogen interactions, proteomics, systems biology

## Abstract

The rapid emergence and global dissemination of antimicrobial resistance (AMR), coupled with the limited development of new antimicrobial agents, have intensified concerns regarding a major global public health crisis. Although antibiotic resistance is primarily driven by microbial mechanisms, host immune and inflammatory responses also play critical roles in determining infection outcomes, pathogen persistence, and treatment efficacy. In this context, host-directed therapies (HDTs) have emerged as a promising complementary strategy by modulating host pathways to enhance antimicrobial immunity, improve bacterial clearance, reduce tissue damage, and restore antibiotic efficacy against multidrug-resistant (MDR) pathogens. Unlike conventional antibiotics, HDTs target conserved host processes, thereby reducing the selective pressure for the emergence of resistance. Recent advances in multi-omics technologies, including genomics, transcriptomics, proteomics, metabolomics, and epigenomics, combined with systems biology approaches, have greatly improved our understanding of host–pathogen interactions during bacterial infections, including those caused by antimicrobial-resistant pathogens. These approaches facilitate the identification of resistance-associated host pathways, molecular networks, and therapeutic targets, while high-throughput screening and computational modelling accelerate the discovery and validation of host-directed interventions. This review highlights emerging host-directed therapeutic strategies against antimicrobial-resistant bacterial infections, with particular emphasis on the use of multi-omics and systems biology to identify and validate host targets associated with resistance phenotypes. We also discuss current approaches to modulate host immune and inflammatory responses, summarize recent advances in host-targeted interventions, and outline the challenges and prospects for translating HDTs into clinical practice to combat AMR.

## 1 Introduction

Antimicrobial resistance (AMR) has become a growing global health challenge with serious long-term impacts (Moriel et al., 2024). It arises when pathogens, including bacteria, viruses, fungi, and parasites, develop resistance to standard treatments, resulting in difficult-to-treat infections caused by so-called “superbugs,” such as methicillin-resistant *Staphylococcus aureus* (MRSA) (Painuli et al., 2023). According to the Global Burden of Disease (GBD) 2021 Antimicrobial Resistance Collaborators, bacterial AMR was associated with an estimated 4.71 million deaths worldwide in 2021, including 1.14 million deaths directly attributable to drug-resistant bacterial infections. While AMR-related mortality has declined substantially among children under 5 years of age over the past three decades, largely due to improvements in vaccination, sanitation, and healthcare, deaths among adults aged ≥70 years have increased by more than 80%, reflecting the growing impact of population ageing and chronic comorbidities (GBD, 2021 Antimicrobial Resistance Collaborators, 2024). A population-level modelling study by Cassini et al. estimated that, in 2015, infections caused by antibiotic-resistant bacteria were responsible for approximately 33,000 attributable deaths and 874,000 disability-adjusted life-years (DALYs) across the European Union (EU) and European Economic Area (EEA) (Cassini et al., 2019). As a result, there is an urgent need to develop new and effective therapies to combat both newly emerging and re-emerging infectious diseases. A comprehensive understanding of the mechanisms responsible for the development and spread of AMR is critical for designing effective therapeutic and preventive strategies. Previous studies on bacterial tolerance and persistence have shown that these adaptive phenotypes can promote the acquisition of antimicrobial resistance through both vertical evolution, involving *de novo* mutations, and horizontal gene transfer mediated by resistance plasmids (Levin-Reisman et al., 2017; Windels et al., 2019; Barrett et al., 2019; Bakkeren et al., 2019). Persister cells represent a small subpopulation of bacteria that enter a dormant or slow-growing physiological state, enabling them to survive under stressful conditions such as antibiotic exposure, oxidative stress, acidic environments, and nutrient deprivation (Kim, 2010). Unlike antibiotic-resistant bacteria, persisters do not undergo permanent genetic changes; rather, their tolerance is transient and reversible. Upon removal of the stress condition, these cells can regrow and remain susceptible to the same antibiotics (Kim, 2007). The ability of persister cells to withstand antimicrobial treatment is strongly associated with chronic, recurrent, and difficult-to-eradicate infections, making them a significant concern in clinical therapeutics (Fisher et al., 2017). Research on the emergence and dissemination of antimicrobial resistance in tolerant and persister bacterial populations is still in its early stages. Nevertheless, accumulating evidence indicates a strong interrelationship among bacterial tolerance, persistence, and antimicrobial resistance, highlighting their interconnected roles in the evolution and spread of resistant infections.

Bacterial infections trigger a highly coordinated host response in which immune signaling, metabolic adaptation, and cellular defense mechanisms function as an integrated and dynamic network rather than as independent pathways. The recognition of pathogen-associated molecular patterns (PAMPs) by pattern recognition receptors (PRRs) initiates inflammatory signaling through NF-κB, MAPK, and interferon pathways, resulting in the production of cytokines and chemokines that recruit and activate innate immune cells. The ensuing inflammatory response increases the metabolic demands of immune cells and, together with infection-induced oxygen depletion, stabilizes hypoxia-inducible factor-1α (HIF-1α), thereby driving immunometabolic reprogramming toward glycolysis to sustain phagocyte activation and antimicrobial functions. Activated phagocytes simultaneously generate reactive oxygen species (ROS), which not only mediate bacterial killing but also function as signaling molecules that amplify cytokine production, regulate inflammasome activation, and influence HIF-1α stability through reciprocal feedback mechanisms. These redox-dependent signals further shape macrophage plasticity, promoting context-dependent activation states that coordinate pathogen clearance, inflammation, tissue repair, and the restoration of immune homeostasis. Conversely, dysregulation of any one of these interconnected pathways can propagate through the network, leading to excessive inflammation, oxidative tissue injury, immune dysfunction, and persistent infection. Owing to this extensive molecular crosstalk, modulation of a single host pathway often exerts pleiotropic effects across multiple immune processes, highlighting the importance of understanding host immunity as a systems-level network rather than as isolated signaling events. Recent advances in multi-omics technologies such as genomics, transcriptomics, proteomics, metabolomics, single-cell sequencing, and spatial omics, combined with systems biology approaches have enabled comprehensive mapping of these interconnected regulatory circuits, facilitating the identification of key host determinants, predictive biomarkers, and therapeutic nodes for host-directed therapies (HDTs). Such an integrated framework provides the mechanistic basis for developing precision HDTs that enhance antimicrobial immunity while minimizing immunopathology and promoting tissue repair.

Host-directed therapies (HDTs) involve approaches that reshape the host’s intracellular, innate, and adaptive immune mechanisms to better manage infectious diseases and associated inflammation. They act either by boosting immune responses to eliminate pathogens or by dampening detrimental immune activity to prevent tissue and organ injury (Wallis and Hafner, 2015). Recently, HDTs have gained attention as a promising strategy for treating microbial infections by modulating the host’s response to pathogens (Schwegmann and Brombacher, 2008). A key difference between HDTs and conventional antibiotics lies in their mechanisms of action. HDTs act by regulating host immune responses and altering host-pathogen interactions, disrupting the mechanisms pathogens rely on for immune evasion and replication. In contrast, antibiotics directly target pathogens to kill them or inhibit their growth. In cases where effective antimicrobial options are limited, such as hepatitis B or AMR bacterial infections, HDTs offer a promising alternative approach (Zumla et al., 2016). By interfering with host pathways that are often evaded by intracellular pathogens, these approaches may offer significant benefits, especially in cases involving drug-resistant organisms where treatment options are limited.

HDTs present several benefits that encourage their use in clinical settings for infection control. Another advantage is their potential to work in synergy with standard antibiotics, supporting combination treatment strategies. Furthermore, HDTs may contribute to reducing the emergence of AMR, as the probability of resistance development is relatively low, particularly when multiple host pathways are involved (Shapira et al., 2024). Host-directed therapies (HDTs) include a wide array of approaches, such as small molecules (Shapira et al., 2024), recombinant cytokines, cell-based therapies, and repurposed drugs, all of which have been investigated for tuberculosis (TB) treatment (Zumla et al., 2016) (Table 1). Strong evidence supports the effectiveness of HDTs against intracellular pathogens, particularly from studies on small-molecule-based strategies that have been comprehensively reviewed (Shapira et al., 2024). Cytokine therapies, especially those utilizing interleukin-2 (IL-2) and interferon-gamma (IFN-γ), have also been widely explored for their immunomodulatory potential (Wallis and Hafner, 2015). Cell-based approaches, including immune and stem cell therapies, provide notable immunomodulatory and anti-inflammatory effects while also contributing to indirect antimicrobial activity (Zumla et al., 2016). In addition, immunotherapies that involve engineering T cells, natural killer (NK) cells, or macrophages with chimeric antigen receptors (CARs) offer a personalised and adaptable strategy for managing chronic and persistent infections (Wallis and Hafner, 2015; Morte-Romea et al., 2024). Repurposed drugs further expand the scope of HDTs and show significant promise as adjunct treatments. For instance, type 2 diabetes mellitus (T2DM) is associated with higher mortality in TB patients, yet the use of metformin has been linked to reduced mortality in TB patients with diabetes (Singhal et al., 2014). Metformin enhances phagolysosome fusion, increases mitochondrial reactive oxygen species (ROS) production, and activates adenosine monophosphate-activated protein kinase (AMPK), thereby promoting autophagy in macrophages and facilitating the clearance of *Mycobacterium tuberculosis* (Wallis and Hafner, 2015; Singhal et al., 2014). Similarly, in the context of HIV–TB co-infection, the antiretroviral protease inhibitor saquinavir has been repurposed for TB management. It improves intracellular killing of *M. tuberculosis*, enhances human leukocyte antigen (HLA) class II presentation in infected macrophages, and promotes T cell activation and proliferation (Pires et al., 2021). Modulating innate immunity as an HDT strategy for treating difficult infections is widely recognised in the field. However, greater emphasis is needed on its clinical implications, particularly the risk of compromising host barrier integrity, with special attention to damage in the intestinal mucosa.

**TABLE 1:** Immunomodulatory host-directed therapeutic strategies against bacterial infections.

Host-directed drug	General mechanism	Pathogen	Development stage	Ref.
Kinase inhibitors				
Imatinib	Inhibits Abl-family and related tyrosine kinases	*M. tuberculosis*	Preclinical	Bruns et al. (2012); Napier et al. (2015); Napier et al. (2011)
Gefitinib	Epidermal growth factor receptor (EGFR) inhibitor; suppresses STAT3 signaling, enhances lysosomal biogenesis and lysosomal targeting of intracellular bacteria	*M. tuberculosis*	Preclinical	Sogi et al. (2017)
Biapenem	Activates the p38 MAPK signaling	*M. tuberculosis*	Preclinical	Pahuja et al. (2023)
Everolimus	Kinase inhibitor	*M. tuberculosis*	Clinical trials	Wallis et al. (2021b)
Targeting lipid synthesis				
Statins (fluvastatin, pravastatin)	HMG-CoA reductase inhibitors	*M. tuberculosis*	Preclinical	Parihar et al. (2014); Dutta et al. (2020)
Atorvastatin	Modulates cholesterol metabolism	*M. tuberculosis*	Preclinical	Parihar et al. (2014)
Etomoxir	Inhibits fatty acid oxidation	*M. tuberculosis*	Preclinical	Huang et al. (2018)
Targeting autophagy				
Nitazoxanide	Stimulates the host’s cellular autophagy	*M. tuberculosis*	Preclinical	Lam et al. (2012)
Pimozide	Inhibits Sirtuin 5 and induces autophagy	*M. tuberculosis*	Preclinical	Heemsker et al. (2021)
Rapamycin	Inhibits mTOR and stimulates autophagy	*M. tuberculosis* *S. aureus*	Preclinical	Bhatt et al. (2021); Gupta et al. (2016); Tasneen et al. (2021); Yu et al. (2023b)
Ambroxol	Induces transcription Factor EB (TFEB)-mediated autophagy	*M. tuberculosis*	Preclinical	Choi et al. (2018)
Metformin	Activates AMPK, inhibits mTOR signaling, and enhances autophagy	*M. tuberculosis*	Preclinical	Singhal et al. (2014)
Tetrandrine	Restores lysosomal acidification and autophagic flux, enhancing intracellular bacterial clearance	*S. aureus*	Preclinical	Lee et al. (2012)
Ar-12	Induces autophagy and modulates PI3K/AKT signaling	*S. aureus*, *S. enterica*	Preclinical	Lo et al. (2014); Chiu et al. (2009b)
Vorinostat (SAHA)	HDAC inhibition	*A. baumannii*	Preclinical	Singothu et al. (2025)
Angiogenesis pathway				
Bevacizumab	Inhibits angiogenesis	*M. tuberculosis*	Preclinical	Lin et al. (2013)
Alisporivir	Binds and inhibits Cyclophilin D	*M. tuberculosis*	Preclinical	Roca and Ramakrishnan (2013)
Wortmannin	PI3K inhibitor; inhibits host phosphatidylinositol 3-kinase (PI3K) activity, disrupting PI3P generation and maturation of the *Salmonella*-containing vacuole (SCV)	*Salmonella enterica* serovar Typhimurium	Preclinical	Scott et al. (2002)
Targeting cAMP and cGMP pathways				
Cilostazol	PDE3 inhibitor; reduces TNF-α by increasing cAMP.	*M. tuberculosis*	Preclinical	Maiga et al. (2013)
Sildenafil	PDE5 inhibitor	*M. tuberculosis*	Preclinical	Maiga et al. (2013)
Pentoxifylline	Nonspecific PDE inhibitor	*M. tuberculosis*	Preclinical	Turner et al. (2001); Wallis et al. (1996)
Thalidomide and their derivates	PDE4 inhibitor	*M. tuberculosis*	Clinical trials	Koo et al. (2011); Subbian et al. (2016a); Subbian et al. (201b); Subbian et al. (2011a); Subbian et al. (2011b)
Targeting macrophage regulation				
Ipilimumab	anti-CTLA-4 monoclonal antibody; promote macrophage polarization	*M. tuberculosis*	Preclinical	Garcia J et al. (2014); Li et al. (2015); Saharia et al. (2016)
IFN-γ	Promotes inflammatory cytokine production, HLA-DR expression, and macrophage activation	*M. tuberculosis*	Clinical trials	Thomas-Rüddel et al. (2025)
CD200-Fc	Regulates CD200-CD200R immune signaling and macrophage activation	*F. tularensis*, *B. pseudomallei*	Preclinical	Casulli et al. (2019); Thom et al. (2023)
Targeting pro-inflammatory responses				
Etanercept	TNF-α inhibitor; reduce inflammation	*M. tuberculosis*	Clinical trials	Moreland, (2004)
MCC950	Selective inhibition of NLRP3 inflammasome activation and IL-1β production	*S. typhimurium,* *M. tuberculosis*	Preclinical	Coll et al. (2015); Subbarao et al. (2020)
Glyburide	Inhibits NLRP3 inflammasome activation and excessive inflammatory responses	*B. pseudomallei*	Preclinical	Koh et al. (2013)
HMGB1-monoclonal antibody	Neutralizes extracellular HMGB1-mediated inflammatory signaling	*B. pseudomallei*	Preclinical	Laws et al. (2015)
Resolvin D1 (RvD1)	Specialized pro-resolving mediator; enhances macrophage phagocytosis, reduces excessive TNF-α/NF-κB inflammation, and restores immune homeostasis	*M. tuberculosis*	Preclinical	Ruiz et al. (2019)
Atorvastatin	Increases biosynthesis of 13-series resolvins through COX-2 S-nitrosylation and enhances resolution of inflammation	*E. coli*	Preclinical	Lee and Zeldin (2015)
Clavanin-MO	Enhances innate immune responses	Multidrug-resistant bacteria including *S. aureus*	Preclinical	Silva et al. (2016)
NewStar2	Activates TNFR2 signaling and modulates immune responses	*S. aureus*	Preclinical	Youn et al. (2023)
Adalimumab and Infliximab	Anti-TNFα blocker monoclonal antibodies	*M. tuberculosis*	Clinical trials	Blackmore et al. (2008); Wallis et al. (2009); Jorge et al. (2012)
Etoricoxib	Cox-2/PGE2 inhibitor	*M. tuberculosis*	Clinical trials	Jenum et al. (2021)
Pascolizumab	Humanized anti-IL-4 monoclonal antibody; blocks IL-4 signaling	*M. tuberculosis*	Clinical trials	Paton et al. (2024)
Others				
Doxycycline	Matrix metalloprotease (MMP) inhibitor	*M. tuberculosis*	Preclinical	Walker et al. (2012)
Rucaparib and Niraparib	Poly (ADP-ribose) polymerase inhibitor	*M. tuberculosis*	Preclinical	van Doorn et al. (2021)
Imipramine	Inhibits host cholesterol trafficking by blocking post-lysosomal cholesterol transport (cationic amphiphilic drug), thereby depriving the pathogen of cholesterol required for intracellular replication	*Anaplasma phagocytophilum*	Preclinical	Xiong et al. (2009)
Roscovitine	Cyclin-dependent kinase (CDK) inhibitor; promotes neutrophil apoptosis, enhances resolution of inflammation, and reduces pulmonary inflammatory injury	*S. pneumoniae*	Preclinical	Hoogendijk et al. (2012)
Dexamethasone	Glucocorticoids and immunosuppressant	*M. tuberculosis*	Clinical trials	Mayanja-Kizza et al. (2005); Critchley et al. (2013); Tobin et al. (2012)

Numerous comprehensive reviews have examined HDTs (Parihar et al., 2019; Tobin, 2015; Frank et al., 2019; Kaufmann et al., 2018), and the small molecules discussed in these works formed the foundation for our search strategy. A recent review discusses emerging HDT strategies to combat *S*. *aureus* infection, particularly in the context of antimicrobial resistance and immune evasion (Zheng et al., 2026). It highlights how *S. aureus* manipulates host immune responses and cellular pathways, including autophagy, inflammatory signaling, and metabolic reprogramming, to survive and persist within the host. However, most of these reviews have concentrated on specific compound classes, such as statins, inhibitors of matrix metalloproteinase or protease (Parihar et al., 2014; Jhilta et al., 2024; Mandal et al., 2025; van den Biggelaar et al., 2024a), or on particular intracellular pathogens, including *M*. *tuberculosis* (Tian et al., 2025; Gholap et al., 2025). Furthermore, the majority of existing reviews have focused on HDTs-mediated enhancement of host immunological and systemic responses, while giving limited attention to innate, cell-intrinsic responses to infection. The present review highlights host-directed therapeutic strategies that target host signaling pathways exploited by bacteria for intracellular invasion, survival, and replication. We also discuss the use of immunomodulators, tool compounds, and high-throughput systems biology approaches to identify novel host targets involved in bacterial pathogenesis.

## 2 Approaches to targeting host inflammatory responses

Although many HDT strategies discussed in this section have been evaluated using laboratory or reference bacterial strains, the host pathways targeted by these interventions, including autophagy, inflammatory signaling, immunometabolism, oxidative stress responses, cell death pathways, and metabolic reprogramming, represent highly conserved components of the host immune system that are activated irrespective of the bacterial AMR phenotype (Figure 1). Unlike conventional antibiotics, which directly target bacterial structures or metabolic processes and are therefore susceptible to resistance mechanisms such as drug inactivation, target modification, or efflux pump-mediated drug extrusion, HDTs primarily modulate host cellular pathways that are exploited by both susceptible and drug-resistant pathogens to establish infection, evade immune surveillance, and persist within host cells. Consequently, mechanistic insights obtained using reference strains provide an important biological foundation for understanding host responses during infections caused by MDR and extensively drug-resistant (XDR) bacteria. For example, inhibition of epidermal growth factor receptor (EGFR) signaling by gefitinib enhances lysosomal biogenesis, promotes phagolysosomal maturation, and restricts intracellular *M*. *tuberculosis* growth by targeting host trafficking pathways rather than bacterial components (Stanley et al., 2014; Sogi et al., 2017). Likewise, pharmacological inhibition of host p38 MAPK signaling using AMG548 induces autophagy and significantly limits intracellular *M. tuberculosis* replication, demonstrating that modulation of host signaling pathways can augment bacterial clearance independently of conventional antimicrobial mechanisms (Stanley et al., 2014). Similar observations have been reported in *S*. *aureus* infections, where host autophagy, inflammatory signaling, and programmed cell death pathways critically regulate intracellular bacterial survival and immune-mediated clearance, including in MRSA infections (Schnaith et al., 2007). In addition, several clinically investigated HDTs, including metformin, vitamin D, and IL-7, have demonstrated the ability to enhance host immune function, improve macrophage antimicrobial activity, restore T-cell responses, and improve treatment outcomes in patients with MDR TB, further supporting the concept that modulation of host immunity represents a resistance-independent therapeutic strategy (Singhal et al., 2014; Wallis RS. et al., 2021). Nevertheless, AMR is frequently associated with alterations in bacterial virulence, metabolism, biofilm formation, persistence, and immune evasion strategies, all of which can reshape host–pathogen interactions and potentially influence the efficacy of HDTs. Emerging transcriptomic, proteomic, metabolomic, and single-cell studies have begun to reveal distinct host immune signatures associated with infections caused by resistant pathogens, highlighting the importance of integrating multi-omics and systems biology approaches to identify resistance-associated host pathways and biomarkers. Therefore, wherever available, this review explicitly highlights evidence obtained using clinically resistant isolates, including MRSA, MDR/XDR *M. tuberculosis*, carbapenem-resistant *Klebsiella pneumoniae*, and multidrug-resistant *Acinetobacter baumannii*. For mechanisms that have thus far been investigated only in reference strains, we discuss their potential relevance to AMR while emphasizing that validation in resistant clinical isolates remains a critical priority for the development and clinical translation of precision host-directed therapies.

**FIGURE 1:**
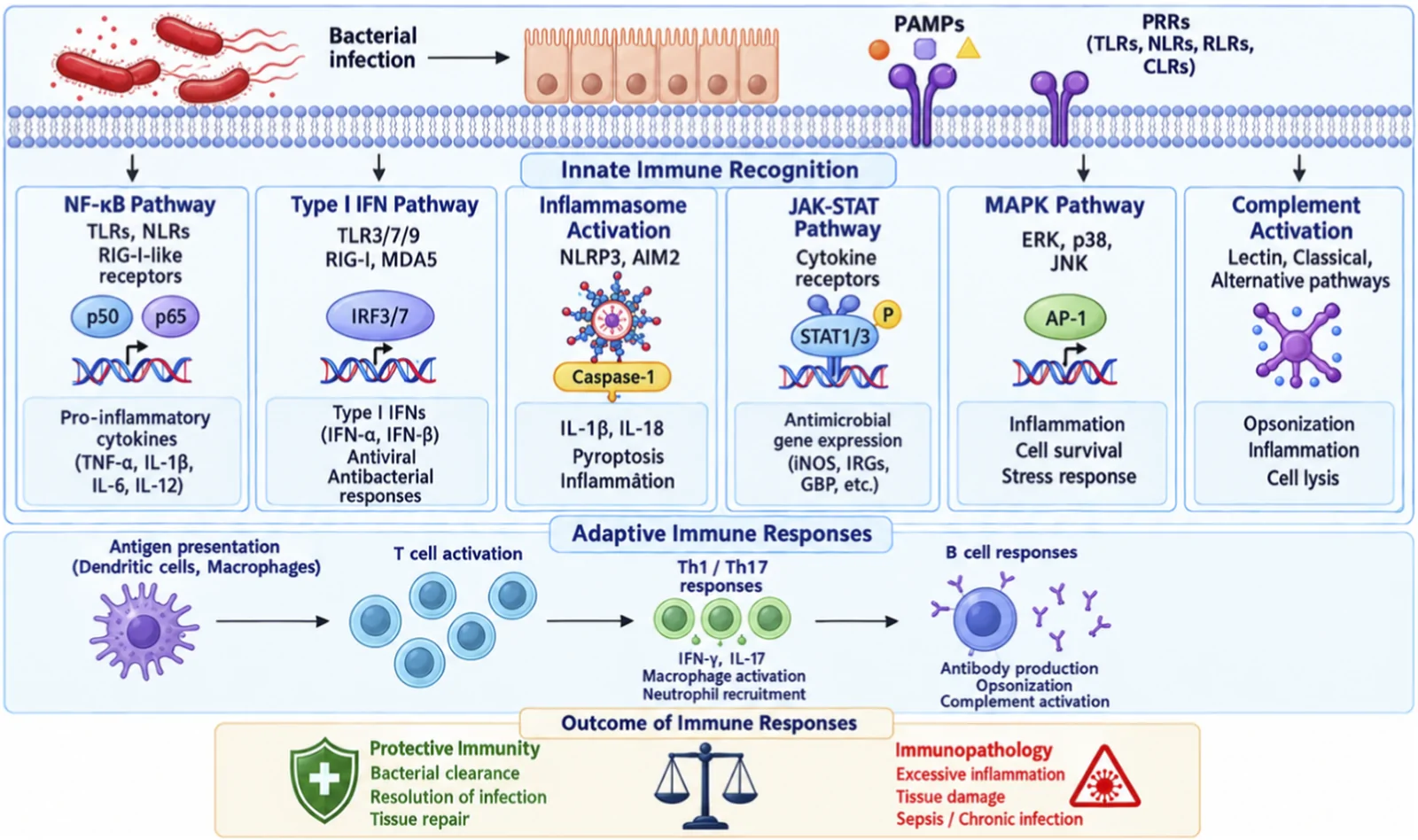
Host immune pathways involved in bacterial infections and their relevance to host-directed therapies (HDTs). Bacterial pathogen-associated molecular patterns (PAMPs) are recognized by pattern recognition receptors (PRRs), triggering key innate immune signaling pathways, including NF-κB, type I interferon (IFN-I), inflammasome, JAK–STAT, MAPK, and complement pathways. These responses promote cytokine production, antimicrobial defense, and antigen presentation, leading to activation of adaptive immunity through T cells and B cells. Balanced immune responses facilitate bacterial clearance and tissue repair, whereas dysregulated activation results in excessive inflammation, tissue damage, and sepsis. These pathways represent major targets for host-directed therapeutic strategies to improve infection outcomes.

### 2.1 Role of autophagy

Autophagy is a cellular mechanism responsible for breaking down cytoplasmic components, such as toxic protein aggregates and damaged organelles, within lysosomes. This process is upregulated in response to cellular stress conditions, including hypoxia, energy depletion, and nutrient scarcity. By recycling internal cellular materials, autophagy supports adaptation to starvation and plays a crucial role in maintaining cellular metabolism and overall homeostasis (Ryter et al., 2013). In addition to maintaining cellular balance, autophagy acts as an innate immune response that removes intracellular pathogens. It begins with the formation of a phagophore, which expands into an autophagosome that encloses cellular material (Figure 2A) (Huang and Brumell, 2014). LC3B is a key protein in the autophagy pathway. It is produced when pro-LC3 undergoes proteolytic processing to form LC3B-II, which helps in autophagosome closure and cargo binding (Mizushima, 2007). The autophagosome then fuses with a lysosome to form an autophagolysosome, where proteases and acid hydrolases degrade the enclosed material (Tjelle et al., 1996). Xenophagy is a specialized form of autophagy that selectively eliminates intracellular microorganisms. It operates through sequestosome 1 (p62)-like receptors or via NOD2-ATG16L1-mediated pathways, which recognize invading pathogens and direct them toward autophagic degradation (Figure 2A) (Deretic, 2012).

**FIGURE 2:**
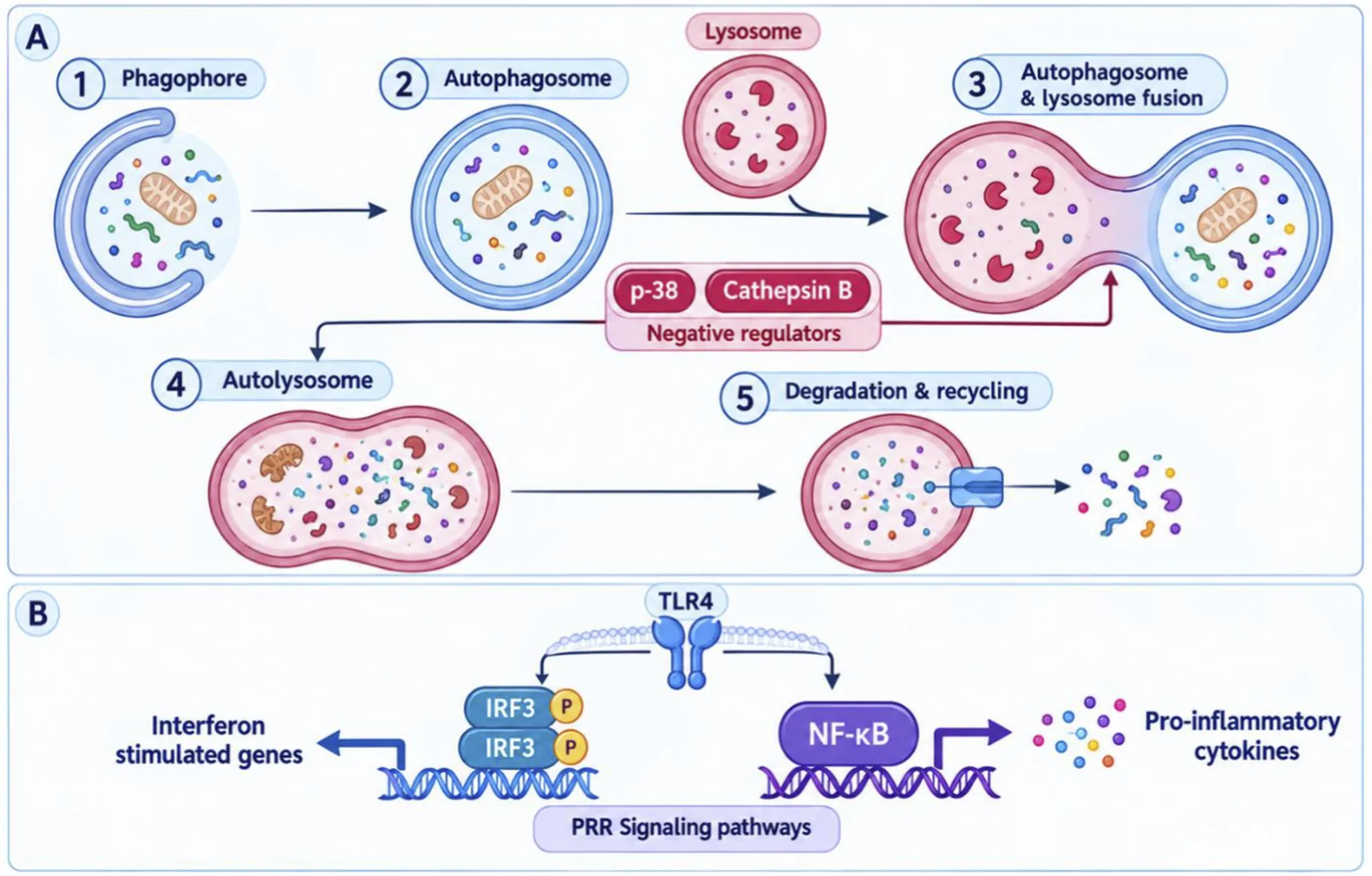
Host-directed therapeutic approaches targeting autophagy and pattern recognition receptor (PRR) signaling pathways to enhance antibacterial immunity. **(A)** Overview of the autophagy pathway, showing phagophore formation, autophagosome maturation, fusion with lysosomes, autolysosome formation, and subsequent degradation and recycling of intracellular cargo. Negative regulators such as p38 MAPK and cathepsin B can impair autophagic flux. **(B)** Activation of PRR signaling through TLR4 engages both the MyD88-dependent and TRIF-dependent pathways. The MyD88 branch activates NF-κB and induces pro-inflammatory cytokines, whereas the TRIF branch activates IRF3, leading to the production of type I interferons and interferon-stimulated genes (ISGs), together promoting antimicrobial host defenses and pathogen clearance.

LC3-associated phagocytosis (LAP) represents an alternative bacterial clearance pathway in which single-membrane phagosomes engulf bacteria and are transiently decorated with LC3-II. This process is activated by various pathogens, including *Escherichia coli*, *Salmonella Typhimurium*, *Mycobacterium marinum*, and *Burkholderia pseudomallei* (Lai and Devenish, 2012). Pharmacological induction of LAP using sirolimus, an mTOR inhibitor, enhances LC3 colocalization with *B. pseudomallei* within phagosomes, promoting phagosomal maturation and reducing intracellular bacterial survival (Rinchai et al., 2015). In contrast, inhibition of autophagy significantly increases intracellular survival of *B. pseudomallei* (Cullinane et al., 2008). However, many intracellular pathogens have evolved strategies to evade autophagic clearance by suppressing autophagy initiation, avoiding recognition by autophagy receptors, disrupting autophagy machinery, or blocking autophagosome–lysosome fusion (Huang and Brumell, 2014). Enhancing autophagy by inhibiting negative regulators of the pathway, such as p38 MAPK and cathepsin B, represents a potential therapeutic strategy for bacterial clearance. p38 MAPK is known to negatively regulate autophagy (Webber and Tooze, 2010). In macrophages, treatment with AMG548, a p38 inhibitor, promotes autophagy and enhances the clearance of *M*. *tuberculosis* (Stanley et al., 2014). Similarly, gefitinib, an epidermal growth factor receptor inhibitor, reduces *M. tuberculosis*-induced p38 phosphorylation, thereby increasing LC3 expression, stimulating autophagy, and limiting bacterial growth (Stanley et al., 2014). Cathepsin B, a lysosomal protease, is involved in the degradation of cellular material as well as immune functions such as TLR activation and antigen processing (Kramer et al., 2017). Cathepsin B-deficient mice infected with *Francisella novicida* show a 40% increase in survival and significantly lower bacterial loads in the liver and spleen compared to wild-type mice (Qi et al., 2016). These findings suggest that cathepsin B inhibitors may serve as potential host-directed antimicrobial therapies. Since these molecules play critical roles in multiple cellular and immune processes, their long-term treatment may lead to unintended effects, including impaired immune regulation, altered inflammatory responses, or off-target toxicity. Furthermore, the efficacy and safety of targeting these pathways in human infections remain insufficiently explored, highlighting the need for comprehensive clinical studies before their therapeutic application can be established.

Intracellular *S. aureus* infection causes metabolic reprogramming in host cells, inducing autophagy that the pathogen exploits for intracellular survival (Vozza et al., 2021). Activation of AMP-activated protein kinase promotes autophagy through ULK1 phosphorylation, replenishing nutrients that inadvertently support bacterial replication. Conversely, inhibition of AMPK disrupts this metabolic adaptation and reduces intracellular bacterial survival. Interestingly, both AMPK activation and inhibition have shown therapeutic potential depending on the stage of infection: activation enhances neutrophil recruitment and bacterial clearance during later stages of infection (Park et al., 2013), whereas early inhibition prevents bacterial exploitation of host resources. These findings showed the context-dependent potential of HDTs (Bravo-Santano et al., 2018).

Inhibition of the host AMP-activated protein kinase pathway by dorsomorphin significantly decreases intracellular survival of MRSA infection by suppressing autophagy (Bravo-Santano et al., 2018). Dorsomorphin blocks AMPK phosphorylation, reduces LC3-I to LC3-II conversion, and impairs autophagic flux, thereby limiting nutrient availability required for bacterial persistence. Studies in infected HeLa cells and human umbilical vein endothelial cells showed reduced autophagy, improved host cell viability, and decreased bacterial survival following treatment, with stronger effects observed in endothelial cells. These findings highlight AMPK inhibition as a promising host-directed therapeutic strategy against intracellular MRSA infection (Bravo-Santano et al., 2018). Certain ATP-competitive kinase inhibitors reduce intracellular MRSA infection by activating AMP-activated protein kinase-mediated autophagy (van den Biggelaar R. et al., 2024). Among compounds screened from PKIS libraries, GW296115X showed potent anti-MRSA activity by enhancing autophagy-dependent bacterial degradation, an effect abolished by inhibition of autophagic flux. Another compound, GW633459A, also reduced intracellular MRSA, although its mechanism remains unclear. Both compounds showed low toxicity in zebrafish models, while GW296115X improved survival in infected embryos (van den Biggelaar R. et al., 2024). This suggests that the role of autophagy in MRSA pathogenesis is highly context-dependent and may vary with cell type, infection stage, and host conditions.

Studies investigating histone modifications during *A. baumannii* infection are limited. One study showed that *A. baumannii* reduces histone acetylation marks (H3K9ac, H3K27ac, and H2AK5ac) and increases HDAC expression in infected THP-1 cells and peripheral blood mononuclear cells (PBMCs), promoting intracellular survival (Singothu et al., 2025). Treatment with the pan-HDAC inhibitor vorinostat (SAHA) restored H3K9ac levels, induced autophagy-related genes, and enhanced autophagosome–lysosome fusion through SNARE proteins, leading to improved intracellular clearance of *A. baumannii* (Singothu et al., 2025). These findings highlight epigenetic modulation as a potential host-directed therapeutic target against multidrug-resistant *A. baumannii*. Despite the promising results, evidence supporting histone modification–based therapies against *A. baumannii* remains limited and is largely derived from a single study using *in vitro* models. As histone deacetylases regulate numerous cellular processes beyond autophagy, broad-spectrum HDAC inhibitors such as vorinostat may produce off-target effects, including altered gene expression, immune dysregulation, and cytotoxicity.

Despite substantial advances in understanding the role of autophagy during bacterial infections, several important knowledge gaps remain. One of the major challenges is the context-dependent nature of autophagy, as its effects vary considerably across bacterial species, infection stages, host cell types, and immune statuses. Another unresolved issue is the limited understanding of the molecular mechanisms governing the interplay between autophagy and other host defence pathways, including apoptosis, inflammasome activation, oxidative stress, metabolic reprogramming, and hypoxia signaling. Although crosstalk among these pathways has been increasingly recognized, the temporal and spatial regulation of these interactions during infection remains poorly characterized. Elucidating these regulatory networks will be essential for identifying optimal therapeutic intervention points. From a translational perspective, most evidence supporting autophagy modulation as a host-directed therapeutic strategy originates from *in vitro* studies and animal models, whereas robust clinical evidence remains scarce. The safety of prolonged pharmacological modulation of autophagy also remains uncertain, as excessive activation may disrupt cellular homeostasis and tissue repair, while sustained inhibition could impair immune surveillance and increase susceptibility to secondary infections. Furthermore, currently available autophagy modulators often exhibit limited specificity because many agents target upstream signaling pathways such as mTOR or AMPK that regulate numerous physiological processes beyond autophagy.

### 2.2 Role of pattern recognition receptor signaling pathways

Pattern recognition receptors (PRRs), including Toll-like receptors (TLRs), NOD-like receptors (NLRs), and RIG-I–like receptors (RLRs), recognize conserved microbial structures known as pathogen-associated molecular patterns (PAMPs). Most TLRs signal through the MyD88-dependent pathway, leading to activation of NF-κB and the subsequent expression of pro-inflammatory cytokines. In contrast, TLR3 signals exclusively through the TRIF-dependent pathway, whereas TLR4 uniquely activates both the MyD88-dependent and TRIF-dependent signaling pathways. Following endocytosis, TLR4 recruits the adaptor proteins TRAM and TRIF, resulting in activation of TBK1/IKKε, phosphorylation of interferon regulatory factor 3 (IRF3), and induction of type I interferons and interferon-stimulated genes (ISGs), in addition to MyD88-mediated NF-κB activation (Figure 2B) (Takeuchi and Akira, 2010). This signaling rapidly induces cytokine and chemokine production, enhancing bacterial clearance. In mouse studies, monophosphoryl lipid A, a detoxified form of lipid A from LPS, significantly reduced nasopharyngeal colonization by *Haemophilus influenzae* and *Moraxella catarrhalis* (Hirano et al., 2011). Moreover, PRR ligands are being developed as vaccine adjuvants; a formulation containing five conserved *S*. *aureus* antigens combined with a TLR7 agonist and alum (4CT 7-Staph) protected against multiple *Staphylococcus* strains in a murine peritonitis model (Bagnoli et al., 2015).

The NLRC4 inflammasome is crucial for controlling several bacterial infections, including those caused by *Salmonella typhimurium*, *Shigella flexneri*, *Pseudomonas aeruginosa*, and *B. pseudomallei* (Matusiak et al., 2015). *B. pseudomallei* activates NLRC4-dependent pyroptosis, a form of programmed cell death that limits intracellular bacterial replication. In contrast, NLRP3 activation drives IL-1β production, which can promote *B. pseudomallei* replication, excessive neutrophil infiltration in the lungs, and subsequent tissue injury (Ceballos-Olvera et al., 2011). In mouse models, glyburide, an IL-1β inhibitor, reduced bacterial (*B. pseudomallei*) spread to the liver and spleen (Koh et al., 2013). However, its therapeutic value in humans remains uncertain due to inconsistent associations between anti-inflammatory effects and clinical outcomes (Liu et al., 2014; Kewcharoenwong et al., 2013; Ko et al., 2011). MCC950, a selective NLRP3 inhibitor that blocks ASC oligomerization without affecting NLRC4 signaling (Coll et al., 2015), also requires further evaluation in the context of bacterial infection. Collectively, selectively targeting NLRP3-mediated cytokine responses represents a promising therapeutic approach. Despite these promising findings, several limitations should be considered. Most evidence supporting the therapeutic targeting of NLRP3 signaling in *B. pseudomallei* infection is derived from animal models and *in vitro* studies, which may not fully recapitulate the complexity of human disease. The protective and pathological roles of inflammasome activation are highly context-dependent, varying with the stage of infection, bacterial burden, and host immune status. In addition, glyburide exhibits pleiotropic effects beyond inflammasome inhibition, making it difficult to attribute its benefits solely to suppression of IL-1β production. Similarly, although MCC950 selectively inhibits NLRP3 signaling, its efficacy, optimal dosing, timing of administration, and long-term safety during bacterial infections remain insufficiently characterized. Furthermore, excessive inhibition of NLRP3-mediated inflammation may impair pathogen clearance and compromise protective immune responses.

Another area of investigation involves the reduction of high mobility group box 1 (HMGB1). HMGB1, a damage-associated molecular pattern (DAMP), promotes pro-inflammatory cytokine signalling and is released by damaged or infected cells; its elevated levels have been linked to poor prognosis in patients with melioidosis (Charoensup et al., 2014). In a *B*. *pseudomallei* mouse model, inhibition of HMGB1 signalling using a monoclonal antibody resulted in a significant decrease in bacterial burden within organ tissues, which was accompanied by reduced pro-inflammatory cytokine levels (Laws et al., 2015). Comparable results were observed in a *Francisella tularensis* mouse model (D’elia et al., 2013), suggesting the potential of these immunomodulatory agents for broad-spectrum therapeutic application.

More recent studies investigating the immunomodulator CD200-Fc have demonstrated its therapeutic efficacy in mouse models of *F. tularensis* LVS (Casulli et al., 2019), as well as in our murine aerosol models of CDC category A priority pathogen, including *B. pseudomallei* (Thom et al., 2023). It suggests that CD200-Fc exerts its effects by binding to its receptor, thereby promoting immune homeostasis through the regulation of Th1 and Th2 cytokine responses, while also enhancing antimicrobial activity via the induction of ROS. This hypothesis is further supported by published findings highlighting the critical role of the CD200 receptor in lung macrophages during severe influenza infection, where it contributes to reduced pulmonary inflammation and the maintenance of immune balance (Snelgrove et al., 2008).

Resolvins, a group of bioactive lipid mediators, have been extensively studied for their ability to promote the resolution of chronic infections and have shown therapeutic potential in a variety of chronic inflammatory conditions, as previously reviewed (Liu C. et al., 2022; Centanni et al., 2023). When used in combination with other HDT approaches described above, resolvins may offer a dual advantage by attenuating disease progression while also safeguarding the host against immunopathological damage.

### 2.3 Interlink between hypoxic and innate immune responses

Bacterial infections commonly occur in hypoxic environments, which can enhance the inflammatory response and aid in clearing the infection (Schaffer and Taylor, 2015). The body’s response to hypoxia is controlled at the transcriptional level by hypoxia-inducible factor (HIF). Hypoxia-inducible factor (HIF) is a heterodimeric transcription factor consisting of an oxygen-regulated α-subunit and a constitutively expressed β-subunit, also known as the aryl hydrocarbon receptor nuclear translocator (ARNT). HIF-1α dimerizes with ARNT to form an active transcription factor that regulates the expression of numerous genes involved in cellular adaptation to hypoxia, including those associated with cytokine production and inflammatory responses. These cytokines play a key role in regulating macrophage aggregation, tissue invasion, and movement. In hypoxia, inactive PHDs allow HIF-1α to accumulate. von Hippel-Lindau tumor suppressor protein (VHL)-deficient macrophages show enhanced killing of *P. aeruginosa*, whereas HIF-1α-deficient macrophages fail to control its replication (Figure 3) (Rius et al., 2008; Peyssonnaux et al., 2005). Mice infected with uropathogenic *E*. *coli* (UPEC) and treated with the HIF-1α stabilizer AKB-4924 showed a 10-fold reduction in bladder colonization, whereas HIF-1α-deficient mice were more susceptible to infection (Lin et al., 2015). These findings suggest that preventing HIF-1α degradation could be a potential therapeutic strategy against bacterial infections.

**FIGURE 3:**
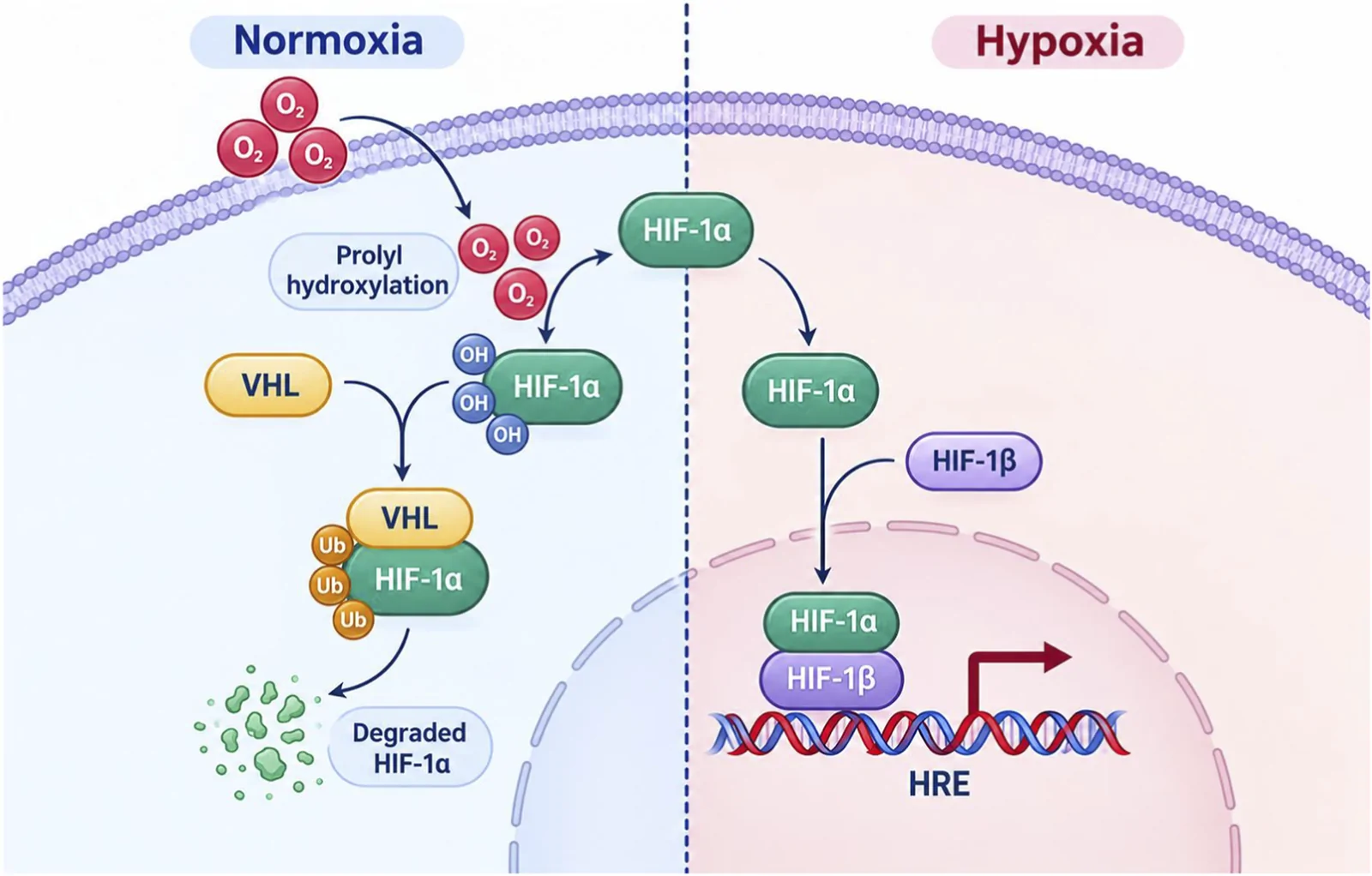
Oxygen-dependent regulation of HIF-1 signaling as a potential host-directed therapeutic target. In normoxia, HIF-1α is hydroxylated and targeted for VHL-mediated degradation. In hypoxia, HIF-1α is stabilized and accumulates in the nucleus, where it forms a heterodimer with HIF-1β (ARNT) and binds hypoxia-response elements (HREs) to activate the transcription of genes involved in metabolism, inflammation, and antimicrobial host defense.

On the other hand, *A. baumannii* engages TLR2 and TLR4, with CD18 acting as a co-receptor, although their exact roles in host defense remain unclear (Morris et al., 2019; Chen, 2020). Activation of transcription factor EB in A549 lung epithelial cells promotes bacterial invasion and intracellular persistence (Parra-Millan and al, 2018). Furthermore, *A. baumannii* induces IL-8 and other pro-inflammatory mediators through NF-κB and MAPK (p38/p44/42) signaling pathways, contributing to inflammatory cell recruitment and activation (March et al., 2010). *A. baumannii* activates NOD-like receptors, with the Nod1/2-Rip2 pathway mediating NF-κB activation and cytokine production, although its contribution to *in vivo* host defense is modest (Kale et al., 2017). TLR9 and the NLRP3-caspase-1/11 pathway also play important roles in host defense and inflammatory cytokine release during infection (Morris et al., 2019; Chen, 2020). While activation of inflammatory pathways such as NF-κB, MAPK, and NLRP3 contributes to bacterial clearance, excessive activation may also promote tissue damage, highlighting the complexity of targeting these pathways therapeutically.

Although pharmacological stabilization of HIF-1α has emerged as a promising HDT strategy to enhance antimicrobial immunity by promoting glycolytic metabolism, phagocyte function, and antimicrobial peptide production, prolonged or uncontrolled HIF-1α activation may result in adverse pathological consequences. Sustained HIF-1α signaling has been associated with metabolic reprogramming, persistent aerobic glycolysis, mitochondrial dysfunction, and disruption of cellular energy homeostasis, potentially impairing tissue repair following infection (Palazon et al., 2014; Taylor and Colgan, 2017). In addition, chronic HIF-1α activation stimulates the expression of profibrotic mediators, including transforming growth factor-β (TGF-β) and connective tissue growth factor (CTGF), thereby promoting extracellular matrix deposition and tissue fibrosis (Higgins et al., 2007). HIF-1α also induces vascular endothelial growth factor (VEGF)-mediated angiogenesis, which, while beneficial for tissue regeneration during acute infection, may contribute to pathological angiogenesis and chronic inflammatory disorders when persistently activated (Palazon et al., 2014). Moreover, prolonged HIF-1α signaling can sustain the production of pro-inflammatory cytokines and delay the resolution of inflammation, thereby increasing the risk of chronic inflammatory pathology (Taylor and Colgan, 2017; Imtiyaz and Simon, 2010). Likewise, although ROS are indispensable for bacterial killing and innate immune defense, excessive ROS production can overwhelm endogenous antioxidant systems, resulting in oxidative damage to lipids, proteins, and nucleic acids, mitochondrial dysfunction, endothelial injury, and exaggerated inflammatory responses that contribute to tissue damage and organ dysfunction during severe bacterial infections (Schieber and Chandel, 2014). Therefore, the clinical success of HIF-1α- and ROS-targeted HDTs depends on achieving a finely regulated balance that enhances antimicrobial immunity while limiting immunopathology and preserving tissue homeostasis.

### 2.4 Role of redox processes in the immune response

Host immune cells, including macrophages and neutrophils, generate ROS and reactive nitrogen species (RNS) as defense mechanisms to promote the clearance of phagocytosed microorganisms. ROS are generated by the host enzyme NADPH oxidase (NOX). The human genome encodes seven NOX homologs, among which NOX2 (also known as gp91phox) is the most well-characterized with respect to its regulatory mechanisms. Pro-inflammatory cytokines such as IL-1β and TNF-α stimulate the expression of inducible nitric oxide synthase (iNOS), an enzyme that generates nitric oxide by converting L-arginine. Bacterial pathogens have evolved mechanisms to evade ROS- and RNS-mediated killing (Figure 4) (Rabadi et al., 2016; Nathan and Shiloh, 2000). *S. typhimurium* blocks the recruitment of phagocyte NADPH oxidase to *Salmonella*-containing vacuoles (Vazquez-Torres et al., 2000), while *F. tularensis* produces antioxidant enzymes, including SodB and SodC, that detoxify ROS and suppress innate immune activation and proinflammatory cytokine production (Rabadi et al., 2016).

**FIGURE 4:**
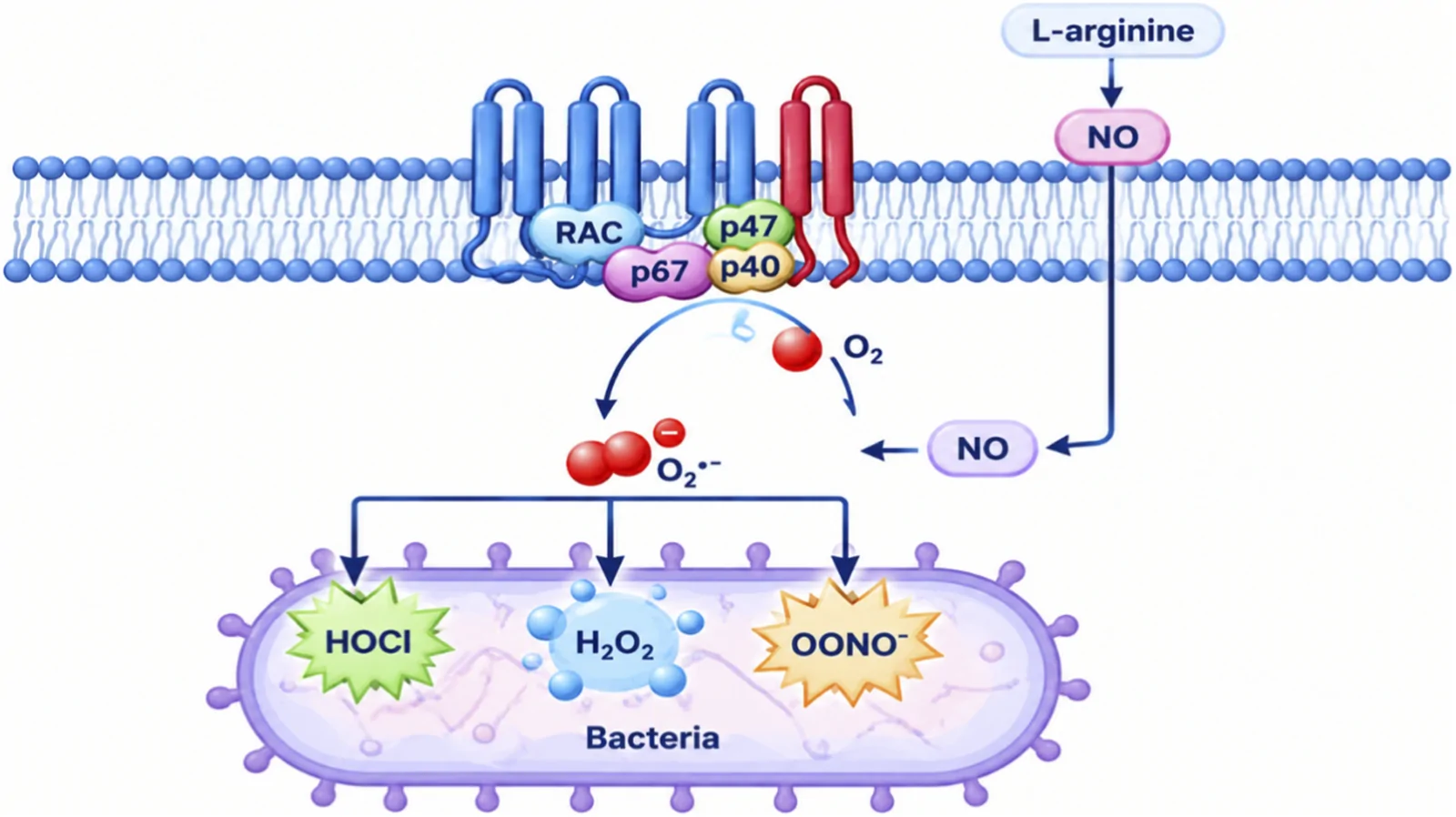
Reactive oxygen species (ROS)- and reactive nitrogen species (RNS)-mediated host-directed antibacterial immunity. Activation of the NADPH oxidase (NOX2) complex at the phagosomal membrane generates superoxide anions (O_2_•^-^), which are subsequently converted into antimicrobial oxidants, including hydrogen peroxide (H_2_O_2_) and hypochlorous acid (HOCl). In parallel, nitric oxide (NO), produced from L-arginine by inducible nitric oxide synthase (iNOS), reacts with superoxide to form peroxynitrite (ONOO^−^), a potent antimicrobial molecule. Together, ROS and RNS contribute to intracellular bacterial killing and represent important host-directed mechanisms for enhancing antimicrobial defense and pathogen clearance.

Defects in ROS production render host cells highly vulnerable to bacterial infections. Chronic granulomatous disease (CGD) is an immunodeficiency caused by impaired NOX2 activity and is associated with increased susceptibility to bacterial infections (Kuhns et al., 2010). In a p47phox-deficient mouse model of CGD, intraperitoneal infection with *Burkholderia cepacia* resulted in mortality, whereas wild-type mice survived, and mutant mice exhibited significantly higher bacterial burdens in the peritoneum, spleen, kidney, and lungs (Pizzolla et al., 2012). In AKB-4929–treated cells, elevated nitrite production was observed and correlated with increased iNOS transcription (Lin et al., 2015). In addition, several anticancer drugs have been reported to increase intracellular ROS levels, although their direct antimicrobial activity has not yet been established (Zhang et al., 2016).

ROS generated by the neutrophil respiratory burst are crucial for killing *S. aureus* and effective innate immune defense (Ha and Edwards, 2021). Defects in NADPH oxidase, as seen in chronic granulomatous disease and knockout mouse models, lead to increased susceptibility to infection (Ha and Edwards, 2021; Pollock et al., 1995), while pharmacological inhibition of the enzyme impairs bacterial clearance (Schieber and Chandel, 2014). Overall, NADPH oxidase–derived ROS are essential for host protection, although low ROS levels may sometimes favor bacterial survival and resistance (Van Acker and Coenye, 2017). HDTs targeting ROS can improve drug specificity while reducing toxicity. Infection-induced redox changes differentiate infected from uninfected cells, as seen in *S. aureus*-infected osteoblasts, which show increased NAD-related activity and elevated NADH/NAD^+^, NADPH/NADP^+^, and glutathione ratios. These shifts can be exploited to develop reduction-responsive therapies for selective targeting of intracellular infections (Ge et al., 2024).

Targeting Tumor necrosis factor receptors represents a promising host-directed approach against *S. aureus* skin infection (Youn et al., 2023). TNFR1 supports neutrophilic abscess formation, while TNFR2 promotes NET formation and ROS production, enhancing antimicrobial defense. Consistently, TNF inhibitor therapy increases susceptibility to *S. aureus* infection. Treatment with the TNFR2 agonist NewSTAR2 reduced skin lesion size and bacterial burden in infected mice by enhancing NET-mediated bacterial clearance, despite lacking direct antibacterial activity (Youn et al., 2023; Vargas et al., 2022).

In the case of *A. baumannii*, neutrophils control through ROS, extracellular traps, and antimicrobial peptides, while macrophages promote early bacterial clearance by recruiting neutrophils and acting synergistically with the complement system (Morris et al., 2019; Chen, 2020; Wong et al., 2017). Drug repurposing studies identified several FDA-approved nonantibiotic drugs with activity against multidrug-resistant pathogens (Andersson et al., 2017). Trifluoperazine, amoxapine, and doxapram improved survival in murine infection models, especially in combination with antibiotics. Additionally, screening identified 121 compounds that reduced *A. baumannii*-induced macrophage cytotoxicity, with six significantly decreasing intracellular bacterial survival (Andersson et al., 2017). These findings support drug repurposing as a promising strategy against multidrug-resistant infections. ROS also play a critical role in killing *Pseudomonas aeruginosa* both *in vitro* and *in vivo* (Sadikot et al., 2004). Mice lacking the p47phox subunit of NADPH oxidase exhibit impaired NF-κB activation and reduced NF-κB–dependent cytokine production, despite enhanced bacterial proliferation (Sadikot et al., 2004). Thus, NADPH oxidase contributes to host defense against *P. aeruginosa* not only through direct microbicidal activity but also by regulating redox-sensitive signaling pathways such as NF-κB in phagocytes.

### 2.5 Attenuating host pro-inflammatory responses

Although inflammatory responses are essential for clearing bacterial infections, systemic infection can trigger excessive inflammation that contributes to sepsis. A class of host-protective lipids known as 13-series resolvins has been shown to enhance bacterial phagocytosis, limit neutrophil recruitment, suppress inflammasome activation, and improve recovery from systemic infection by promoting resolution of inflammation (Lee and Zeldin, 2015). In mice infected intraperitoneally with *E. coli*, resolvin administration increased survival by approximately 40%, without exhibiting direct bactericidal or bacteriostatic effects. Additionally, systemic treatment with atorvastatin enhanced resolvin biosynthesis via S-nitrosylation–mediated activation of endothelial COX-2, thereby accelerating infection resolution and improving survival in *E. coli*-infected mice (Dalli et al., 2015).

Clavanin-MO, a synthetic derivative of clavanin A, exhibits both antimicrobial and immunomodulatory activity against multidrug-resistant *E. coli* and methicillin-resistant *S. aureus* (Silva et al., 2016). It increases IL-10 while suppressing TNF-α and IL-12 in response to LPS, and *in vivo* treatment after *E. coli* infection resulted in 80% survival (Silva et al., 2016), highlighting its ability to reduce inflammation and enhance host survival.

MDR *K. pneumoniae* is a major opportunistic pathogen associated with high mortality in hospitalized and immunocompromised patients due to its remarkable ability to acquire antibiotic resistance traits (Poerio et al., 2022). In an *in vitro* human macrophage model, a combined host- and pathogen-directed therapy using phosphatidylinositol 5-phosphate-loaded apoptotic body-like liposomes (ABL/PI5P) and the lytic bacteriophage φBO1E effectively reduced both intracellular and extracellular bacterial loads (Poerio et al., 2022). The treatment also modulated inflammation by decreasing TNF-α and IL-1β levels while enhancing anti-inflammatory TGF-β production (Poerio et al., 2022). These findings support combined host- and pathogen-directed approaches as promising alternatives for managing MDR-*K. pneumoniae* infections.

### 2.6 Targeting macrophage polarization

Although macrophages typically acquire an M1 phenotype during infection to facilitate pathogen clearance, several pathogens can manipulate host immune responses by driving macrophages toward an M2-polarized state, thereby promoting immune evasion and persistence (Shi et al., 2019). Among the contributing factors, ESAT-6 has been reported to play a key role in inducing M2 polarization and suppressing protective inflammatory responses (Shi et al., 2019). Consequently, targeting macrophage polarization pathways has emerged as a promising strategy to limit pathogen survival and disease progression (Bouzeyen et al., 2019; Singh VK. et al., 2022). Another HDT approach aims to promote M1 macrophage polarization via activation of the p38 MAPK pathway. Biapenem and withaferin A have been shown to enhance pro-inflammatory responses, synergize with rifampin and isoniazid, and reduce TB relapse rates (Kumari et al., 2023; Pahuja et al., 2023). Notably, withaferin A significantly lowered MDR-*M*. *tuberculosis* burden in infected mice (Pahuja et al., 2023). Similarly, SQ109 induces pro-inflammatory signaling through p38 MAPK activation (Singh M. et al., 2022), while rocaglates enhance IFN-γ–mediated M1 polarization and inhibit ERK1/2 and Myc signaling associated with M2 polarization (Chatterjee et al., 2021). Additionally, activating transcription factor 2 (ATF2) has emerged as a potential HDT target for modulating macrophage polarization (Rajabalee et al., 2023). Additionally, the identification of an autoregulatory feedback loop between ATF2 and PPM1A has highlighted the role of PPM1A inhibition in host defense against *M*. *tuberculosis* (Rajabalee et al., 2023).

Another study demonstrated that both natural and synthetic rocaglates promote M1-like macrophage activation by increasing sensitivity to low levels of IFN-γ and inhibiting IL-4-mediated M2 polarization (Chatterjee et al., 2021). Rocaglates are a group of bioactive compounds derived from medicinal plants belonging to the *Aglaia* genus, traditionally used in Chinese medicine to treat fever, cough, diarrhea, and inflammation. Furthermore, rocaglates enhanced phagosome–lysosome fusion, leading to improved intracellular mycobacterial clearance in primary macrophages (Chatterjee et al., 2021). The macrophage-activating effects of rocaglates, both IFN-dependent and independent, were partly mediated through activation of the p38 MAPK pathway. Rocaglates induced the expression of p62, Txnip, and Ptgs2 in naïve bone marrow–derived macrophages in a p38-dependent manner and synergized with IFN-γ by promoting non-canonical p38-mediated phosphorylation of STAT1, thereby enhancing macrophage activation (Chatterjee et al., 2021). Rocaglates suppress alternative (M2-like) macrophage activation by inhibiting STAT6 transcriptional activity through a p38-independent mechanism. They also target anabolic and pro-survival pathways by downregulating CSF1 receptor and Myc expression, while inhibiting ERK1/2 phosphorylation. Since ERK-mediated Myc activation is essential for the induction and maintenance of the M2 phenotype, rocaglate-mediated ERK inhibition likely disrupts Myc-dependent pathways involved in M2 polarization (Pello, 2016; Pello et al., 2012). Therefore, the potent inhibition of ERK phosphorylation by rocaglates likely contributes significantly to the suppression of both STAT6- and Myc-dependent signaling pathways, thereby inhibiting the induction and maintenance of the M2-like macrophage phenotype.

Similarly, during *S. aureus* infection, M2 macrophage polarization displays dual roles depending on the duration and stage of the immune response. In the acute inflammation resolution phase, M2 macrophages attenuate excessive inflammatory signaling by producing IL-10, TGF-β, and Arg-1, which helps prevent tissue injury while promoting angiogenesis, fibroblast activation, and tissue repair (Banerjee et al., 2024). Emerging evidence in immunometabolism suggests that M2 macrophage activity is supported by oxidative phosphorylation and fatty acid oxidation, which facilitate sustained tissue repair functions. In MRSA skin and soft tissue infection models, myeloid GLUT1 promoted anti-inflammatory macrophage polarization via a PPARγ-dependent pathway, resulting in accelerated infection resolution and improved host recovery (Banerjee et al., 2024). Furthermore, specialized pro-resolving mediators (SPMs), such as resolvins and maresins, contribute to apoptotic cell clearance and enhance anti-inflammatory responses, thereby reducing immunopathology and supporting tissue regeneration during infection (Jordan et al., 2020; Miek et al., 2022). Peng et al. reported that *S*. *aureus* infection biofilms, in an implant infection model, promoted mesenchymal stem cell (MSC) proliferation and skewed their differentiation toward an immunosuppressive M2-like phenotype, thereby supporting persistent bacterial survival (Cavallo et al., 2024; Peng et al., 2017). Consistently, Thurlow et al. demonstrated in a murine skin abscess model that biofilm-associated infection drives M2 macrophage polarization, disrupts effective inflammatory containment, and favors chronic infection (Thurlow et al., 2011). The immunomodulatory functions of MSCs are primarily mediated through exosomes, which act as key vehicles for intercellular communication by transporting bioactive molecules such as regulatory microRNAs (Zhang et al., 2024). These exosomes can attenuate M1-driven inflammatory responses through suppression of the MyD88/STAT1 signaling pathway and enhancement of CD73-dependent adenosine signaling, thereby promoting M2 macrophage polarization and supporting anti-inflammatory and tissue repair processes (Lv and Liang, 2025; Teo et al., 2023). Importantly, *S. aureus* infection also produces extracellular vesicles capable of disrupting macrophage efferocytosis, which may hinder wound repair and contribute to persistent infection (Ou et al., 2025). Recent studies suggest that esculin, a metabolite derived from the sauerkraut-associated bacterium *Bacillus safensis* SK14, modulates immune responses via the gut–lung axis in MRSA-induced acute lung injury. Mechanistically, esculin inhibits TLR2–MyD88–NF-κB signaling while activating the Nrf2-ARE pathway, leading to suppression of M1 polarization, enhancement of M2 polarization, reduced inflammation, and improved antioxidant capacity (Pan et al., 2025). Likewise, esculetin exerts anti-inflammatory effects in sepsis-induced acute lung injury by inducing metabolic reprogramming of macrophages, suppressing M1 glycolysis, and promoting M2-associated fatty acid β-oxidation to restore polarization homeostasis (Chen et al., 2024). However, the long-term consequences of promoting M2 polarization are not fully understood, as excessive M2 activation may impair host defense or contribute to fibrosis. Further studies are needed to establish optimal dosing, pharmacokinetics, and clinical applicability of these compounds. In addition to compounds that induce M1 polarization, some agents enhance macrophage antimicrobial activity independent of phenotype. PARP inhibitors such as Rucaparib and Niraparib reduced intracellular burdens of drug-susceptible and MDR *M. tuberculosis* in infected macrophages by modulating cytokine and surface marker expression (van Doorn et al., 2021). However, they increased bacterial loads in M2 macrophages derived from healthy donors, highlighting the variability of human macrophage responses (van Doorn et al., 2021).

## 3 Multi-omics identification of host targets in bacterial infection

The term multi-omics refers to the integration of different -omics platforms to achieve a more comprehensive and reliable interpretation of biological phenomena, improving the robustness of conclusions drawn from single-omics analyses. The rapid emergence of MDR, XDR, and pandrug-resistant (PDR) bacterial pathogens has shifted the focus of antimicrobial research from pathogen-centric approaches toward a more comprehensive understanding of host-pathogen interactions. While bacterial resistance is traditionally attributed to genetic mechanisms such as target modification, enzymatic antibiotic degradation, efflux pump activation, horizontal gene transfer, and biofilm formation, accumulating evidence demonstrates that host immune responses also profoundly influence bacterial persistence, treatment efficacy, and clinical outcomes. Importantly, AMR is increasingly recognized not only as a bacterial trait but also as a complex systems-level phenomenon involving dynamic interactions between bacterial adaptation and host immune regulation. Consequently, comprehensive characterization of host responses during resistant infections has become a major research priority for identifying biomarkers of disease progression, understanding mechanisms of treatment failure, and discovering novel HDT targets. The central aim of this framework is to provide a more complete representation of biological systems, thereby reducing the risk of incorrect hypothesis formation and misinterpretation of complex data. In essence, -omics-based methodologies are fundamentally systems-level approaches by design. Recent advances in high-throughput technologies, including transcriptomics, proteomics, metabolomics, and imageomics, have created powerful opportunities to systematically uncover host–pathogen interactions and define gene functions during bacterial infection. Next-generation sequencing technologies have greatly expanded the scope of integrated genomics–transcriptomics and epigenomics–transcriptomics research through a wide range of “-seq” approaches. These include whole-genome sequencing, exome sequencing, Methyl-seq, DNase-seq, ATAC-seq, ChIP-seq, RNA-seq, Ribo-seq, and polysome profiling, all of which enable detailed, genome-wide analysis of gene regulation and expression. Alongside these advances, mass spectrometry (MS)-based proteomics has become a key platform for investigating host-pathogen interactions by allowing comprehensive quantification of protein abundance, post-translational modifications, secretion dynamics, and protein-protein interactions. Metabolomics complements these approaches by enabling large-scale profiling of metabolites, thereby capturing rapid and dynamic metabolic shifts that occur in response to physiological stresses, including bacterial infections.

Host kinases act as key regulators in bacterial infection by controlling essential signaling pathways that pathogens often exploit to facilitate entry, survival, immune evasion, and intracellular replication. The AKT1 signaling pathway is a key regulator of host immune responses across diverse bacterial infections and has therefore emerged as an important target for host-directed antibacterial therapies (Krachler et al., 2011). A siRNA kinome screen demonstrated that *S*. *typhimurium* secretes the effector protein SopB, which interferes with phagosome–lysosome fusion by suppressing AKT1 activation in host cells (Kuijl et al., 2007). In addition, phosphatase-targeted siRNA screening in *S. typhimurium*- and *M. tuberculosis*-infected host cells showed that nearly 50% of the identified phosphatases were associated with AKT-related signaling networks (Albers et al., 2014). During *P*. *aeruginosa* infection, PI3K-mediated generation of phosphatidylinositol (3,4,5)-trisphosphate promotes recruitment of AKT1, thereby facilitating bacterial transcytosis (Kierbel et al., 2007). Conversely, *K. pneumoniae* activates an anti-inflammatory pathway involving AKT1 that downregulates NF-κB signaling, contributing to immune evasion (Fran et al., 2013). Together, these findings showed the central role of the PI3K/AKT1 axis in regulating intracellular bacterial survival, making it a promising therapeutic target for intervention. The small-molecule AR-12, a non–COX-2 inhibitory derivative of celecoxib, targets this pathway by inhibiting PDK-1 phosphorylation, which consequently reduces AKT1 activation and suppresses PI3K/AKT signaling (Zhu et al., 2004). AR-12 has also been reported to induce autophagy, further contributing to its antimicrobial effects (Chiu et al., 2009a; Lo et al., 2014). In murine models of *S. typhimurium* infection, oral treatment with AR-12 reduced bacterial burden in the spleen and liver by over 90%, delayed disease progression, and improved survival outcomes (Chiu et al., 2009b). Moreover, AR-12 enhances the activity of aminoglycoside antibiotics against intracellular *S. typhimurium* both *in vitro* and *in vivo* (Chen et al., 2024). It has also been shown to inhibit intracellular survival of *Francisella novicida* and *Francisella tularensis* in cell-based assays (Chiu et al., 2009b). Although antibiotic resistance in *F. tularensis* has not yet been widely documented, continued exposure to agents such as aminoglycosides and fluoroquinolones may increase the risk of resistance emergence (Sutera et al., 2014).

Non-receptor tyrosine kinases and inositol-requiring enzyme 1 (IRE1β) are increasingly recognized as host factors that bacteria exploit during invasion, intracellular replication, and survival within host cells (Pielage et al., 2008; Qin et al., 2008). Non-receptor tyrosine kinase signaling, in particular, plays a pivotal role in bacterial internalization by coordinating actin cytoskeletal remodeling and downstream signaling cascades. Through a dsRNA-based functional screen targeting conserved actin-regulatory genes, Pielage et al. (2008) demonstrated that *P. aeruginosa* entry into host cells requires a tightly regulated signaling module comprising Abl tyrosine kinase, the adaptor protein Crk, and the small GTPases Rac1 and Cdc42, along with p21-activated kinase (Pielage et al., 2008). In parallel, IRE1β, a conserved transmembrane kinase involved in the host unfolded protein response, was identified via an RNA interference (RNAi) screen as a critical host determinant required for *Brucella melitensis* infection, underscoring its role in facilitating bacterial intracellular establishment and persistence (Qin et al., 2008).

Recent advances in single-cell RNA sequencing (scRNA-seq), single-cell multi-omics, and spatial transcriptomics have revolutionized the study of host–pathogen interactions by enabling high-resolution characterization of individual immune cells while preserving their spatial organization within infected tissues. These technologies have uncovered remarkable cellular heterogeneity, revealed distinct immune-cell states associated with bacterial persistence, and identified novel host pathways that represent promising targets for HDTs, particularly in infections caused by MDR pathogens (Stubbington et al., 2017; Papalexi and Satija).

Several independent investigations have revealed remarkable heterogeneity among pulmonary and circulating immune cells during *M*. *tuberculosis* infection. Single-cell profiling of PBMCs from patients with active TB demonstrated expansion of inflammatory monocyte populations characterized by elevated expression of S100A8, S100A9, FCN1, IL1B, and CXCL8, together with depletion of cytotoxic NK cells and functional CD8^+^ T lymphocytes (Wang et al., 2023). These transcriptional signatures correlate closely with disease severity and bacterial burden, suggesting that dysregulated innate immunity contributes to persistent infection. More importantly, recent studies involving patients with MDR and XDR TB indicate that persistent bacterial infection is accompanied by profound remodeling of host immune-cell composition. scRNA-seq analyses have identified inflammatory macrophage subsets exhibiting increased expression of IL-1β, NLRP3, CXCL10, and interferon-stimulated genes, whereas macrophages associated with successful bacterial clearance preferentially express genes involved in antigen presentation, oxidative phosphorylation, and phagolysosomal maturation. These findings suggest that MDR-TB is associated not only with bacterial drug resistance but also with maladaptive host inflammatory responses that promote bacterial persistence and treatment failure (Singhania et al., 2018). Single-cell analyses have also identified multiple dysfunctional T-cell populations during chronic TB. Expanded populations of exhausted CD4^+^ and CD8^+^ T cells expressing immune checkpoint molecules including PDCD1 (PD-1), LAG3, HAVCR2 (TIM-3), and TIGIT have been observed in active disease, indicating progressive impairment of protective cellular immunity. These observations provide a mechanistic rationale for exploring immune checkpoint modulation as an adjunctive host-directed therapeutic strategy in drug-resistant TB (Chang et al., 2025).

Although most single-cell studies have focused on TB, rapid progress has also been made in understanding host responses to antimicrobial-resistant Gram-positive and Gram-negative pathogens. RNA-sequencing (RNA-Seq) analyses of *S. aureus* infection have revealed marked heterogeneity among neutrophils, monocytes, and macrophages. Inflammatory neutrophil populations enriched for S100A8, S100A9, CXCL8, and other antimicrobial effector genes coexist with macrophage populations exhibiting distinct inflammatory or tissue-repair phenotypes, highlighting the complexity of host immune responses during staphylococcal infection (Zwack et al., 2022).

Emerging single-cell transcriptomic studies have begun to characterize host immune responses during carbapenem-resistant *K. pneumoniae* (CRKP) infection. scRNA-seq analysis of peripheral blood immune cells from patients with CRKP pneumonia demonstrated extensive immune remodeling characterized by enhanced interferon responses and inflammatory activation across multiple immune-cell populations (Dumigan et al., 2022). The study further identified distinct transcriptional signatures in monocytes, macrophages, and NK cells, including increased expression of S100A12 in monocytes/macrophages and C1QB in macrophages, suggesting that innate immune dysregulation contributes to disease progression. These findings highlight the potential of single-cell transcriptomics to identify host pathways that may be exploited for host-directed therapeutic intervention during CRKP infection (Dumigan et al., 2022). Using single-cell RNA sequencing (scRNA-seq) of peripheral blood mononuclear cells from patients with CRKP pneumonia, Sun et al. identified extensive immune remodeling characterized by inflammatory activation of monocytes and macrophages, with increased expression of S100A12, IFITM3, ISG15, IFI6, IFI27, and the macrophage marker C1QB. Pathway enrichment analysis revealed activation of type I interferon signaling, cytokine-mediated immune responses, antigen presentation, and leukocyte activation, while cell–cell communication analysis demonstrated coordinated interactions among monocytes, macrophages, NK cells, and T lymphocytes. These findings highlight the ability of single-cell transcriptomics to uncover host immune signatures and conserved pathways that may serve as biomarkers and potential host-directed therapeutic targets for combating carbapenem-resistant bacterial infections.

One of the most significant findings from this multi-omics investigation was the identification of inosine monophosphate dehydrogenase 2 (IMPDH2) as a critical host factor supporting *B. abortus* intracellular growth. IMPDH2 catalyzes the rate-limiting step in *de novo* guanine nucleotide biosynthesis, a pathway exploited by *Brucella* to satisfy the increased nucleotide demand associated with intracellular replication. Functional validation using genetic silencing and pharmacological inhibition demonstrated that suppression of IMPDH2 significantly reduced intracellular bacterial replication in macrophages and markedly decreased bacterial burden in infected mice, confirming that host nucleotide metabolism represents a viable therapeutic target independent of bacterial resistance mechanisms. Importantly, subsequent screening of a natural-product library identified oxymatrine, a quinolizidine alkaloid isolated from *Sophora* species, as a selective host-directed inhibitor of IMPDH2. Treatment with oxymatrine effectively suppressed *B. abortus* replication both *in vitro* and *in vivo* without directly targeting bacterial viability, illustrating the therapeutic potential of targeting host metabolic pathways rather than bacterial components (Yu J. et al., 2023).

Reverse-phase protein microarray (RPMA)–based studies have facilitated the identification of host targets by enabling high-throughput profiling of changes in protein expression and phosphorylation during bacterial infection. Using this approach, modulation of host signaling pathways was assessed in extracts from RAW 264.7 macrophages infected with multiple *Burkholderia pseudomallei* strains at different post-infection time points. The detection of phosphorylated AMPK-α1, Src, and glycogen synthase kinase 3β (GSK-3β) highlighted the involvement of metabolic regulation and innate immune signaling pathways in the host response to *Burkholderia* infection (Chiang et al., 2015).

Additionally, integrative analyses using targeted metabolomics and high-throughput proteomics revealed infection-associated alterations in metabolites and proteins within mucosal tissues from chinchillas with nontypeable *Haemophilus influenzae* (NTHi)-induced acute otitis media. Functional validation demonstrated that inhibition of the actin-related Arp2/3 complex in human epithelial cells significantly impaired NTHi infection, confirming its role as a critical host factor (Harrison et al., 2016). Collectively, these studies provide important insights into host–pathogen interaction networks and offer a foundation for therapeutic strategies aimed at disrupting key host–pathogen protein complexes.

A multi-omics study integrating proteomic, transcriptomic, and metabolomic analyses of Brucella-infected patients, mice, and cells generated a comprehensive map of host physiological responses to infection (Yu J. et al., 2023). The study identified key metabolic pathways, cellular responses, and molecular mechanisms involved in disease progression. The analysis revealed that *Brucella* enhances host nucleotide synthesis to support its increased biomass demands. Notably, IMPDH2 was identified as a critical regulator of nucleotide biosynthesis during infection. Pharmacological inhibition of IMPDH2, the rate-limiting enzyme in guanine nucleotide synthesis, effectively suppressed *B. abortus* growth *in vitro* and *in vivo*. Screening of natural products further identified oxymatrine, an alkaloid derived from Sophora species, as a selective IMPDH2 inhibitor (Yu J. et al., 2023).

## 4 Using systems biology to identify host targets involved in bacterial infection mechanisms

Systems biology is a broadly defined approach to biological research that focuses on hypothesis generation and seeks to understand the dynamic interactions and relationships among molecular processes at the level of the entire organism or system. It combines computational and mathematical modeling with diverse -omics technologies to generate large-scale datasets that simultaneously capture multiple aspects of cellular function and regulation. A complementary approach for dissecting host–pathogen interactions is the construction of protein-protein interaction (PPI) networks between host factors and bacterial virulence proteins. Using a yeast two-hybrid (Y2H) library comprising both human and murine proteins, Memišević et al. (Memiševic´ et al., 2013) mapped host interactors of multiple *Burkholderia mallei* virulence factors and identified a highly connected interaction network, including several previously uncharacterized bacterial proteins, thereby underscoring their potential contribution to *B. mallei* pathogenicity and validating the biological relevance of the interactions detected (Memiševic´ et al., 2013). In a similar Y2H-based study, Yang et al. showed that *Yersinia pestis* infection engages host pathways associated with focal adhesion dynamics, cytoskeletal regulation, leukocyte transendothelial migration, and key immune signaling cascades such as TLR and MAPK pathways (Yang et al., 2011). Further, Schweppe et al. (2015), employing chemical cross-linking combined with large-scale mass spectrometry, identified interactions between *Acinetobacter baumannii* virulence determinants and host structural proteins involved in cell–cell adhesion, highlighting mechanisms that support bacterial colonization and dissemination during infection (Schweppe et al., 2015).

The outcome of Mtb infection is governed by complex molecular interactions between bacterial and host proteins, forming a dynamic host–pathogen protein–protein interaction (PPI) network. To elucidate these interactions, a network-based approach was employed to analyze Mtb intra-species protein interactions using Louvain community detection and to investigate inter-species interactions between Mtb and human proteins. The study demonstrated that the Mtb intra-species PPI network exhibited higher connectivity compared with the host–pathogen interaction network, suggesting that bacterial protein network organization plays a crucial role in adaptation and pathogenicity. Through an interolog-based prediction strategy, analysis of 269 Mtb proteins and 2287 human proteins identified 640 host–pathogen interaction pairs involving 90 Mtb proteins and 120 human proteins. These interactions were further prioritized based on functional annotation and experimental validation data. Network analysis of Mtb proteins identified Rv1286, Rv0462, Rv2215, Rv3559c, Rv3504, and Rv3523 as important network motifs and potential regulatory hubs. This comprehensive interactome-based approach enhances understanding of the molecular mechanisms underlying Mtb-host interactions and provides a framework for identifying critical proteins associated with TB pathogenesis and potential therapeutic intervention (Verma et al., 2022).

Integrated genomics–transcriptomics approaches are increasingly employed to unravel host–pathogen interactions and have substantially advanced understanding of how host genetic variation shapes immune responses. In particular, these studies have characterized cis-eQTLs, which regulate expression of nearby genes, and trans-eQTLs, which influence distal gene expression, often across chromosomes, as key genetic determinants of infection-induced transcriptional programs. Lee et al. (2014) profiled human dendritic cells from individuals stimulated with *E*. *coli* LPS, influenza virus, or IFN-β by integrating BeadChip-based genotyping with gene microarray and NanoString transcriptomic analyses. This work identified 121 cis-acting genetic variants associated with variability in stimulus-dependent transcriptional responses (Lee et al., 2014).

In a complementary study, Orozco et al. analyzed macrophages derived from 92 strains of the Hybrid Mouse Diversity Panel using gene microarray-based transcriptomics across genetically characterized inbred mouse strains. Following stimulation with lipopolysaccharide (LPS) or oxidized phospholipids, the authors identified thousands of expression quantitative trait loci (eQTLs) and demonstrated that 2310061C15Rik functions as a novel regulator of macrophage inflammatory responses (Orozco et al., 2012). Similarly, transcriptomic and genotypic profiling of LPS-stimulated human monocytes identified 1,471 eQTLs associated with genes involved in innate immune signaling, chemokine regulation, epigenetic control, and interferon responses, illustrating how host genetic variation shapes inflammatory pathways during bacterial infection (Kim et al., 2014). Expanding upon these findings, a larger population-based study integrating transcriptomic profiling with eQTL mapping following IFNγ and LPS stimulation revealed extensive cis- and trans-regulatory networks involving the major histocompatibility complex (MHC), type I interferon signaling, and interferon regulatory factor (IRF)-dependent transcriptional programs. Integration with ChIP-seq analysis further validated IRF2-mediated regulatory interactions, demonstrating the power of combining transcriptomics, genomics, and epigenomics to identify host pathways that govern innate immune responses (Fairfax et al., 2014).

Host defense is further regulated through dynamic interactions between cytokine signaling, TLR activation, and epigenetic remodeling that collectively shape transcriptional responses during infection. Using an integrated multi-omics approach, Park et al. (2017) combined RNA-seq, ATAC-seq, and ChIP-seq to investigate chromatin accessibility, histone modifications, transcription factor binding, and gene expression in human macrophages following LPS stimulation with or without TNF pretreatment. Their analysis demonstrated that TNF-induced immune tolerance selectively suppressed inflammatory gene programs while largely preserving metabolic and antiviral responses. Importantly, type I interferons reversed this tolerant phenotype, highlighting the coordinated regulation of chromatin remodeling and cytokine signaling during macrophage activation (Park et al., 2017). Complementing these findings, time-resolved RNA-seq and ChIP-seq analysis of LPS-stimulated mouse dendritic cells revealed that multiple transcription factors are pre-positioned at regulatory chromatin regions before gene activation, enabling rapid and coordinated induction of immune-response genes following bacterial stimulation (Garber et al., 2012).

Bottom-up mass spectrometry (MS)-based proteomics has enabled high-resolution profiling of proteomes, phosphoproteomes, and kinomes, providing a powerful framework for dissecting host–pathogen interactions (Khan et al., 2018a; Aebersold and Mann, 2016; Boersema et al., 2015; Manes and Nita-Lazar, 2018). In parallel, MS-based workflows have been widely adapted to additional -omics layers, including secretomics and lipidomics, further expanding their utility in infection biology research (Khan et al., 2018b; Koppenol-Raab et al., 2017). These proteomic strategies have been extensively applied to investigate TLR-mediated innate immune signaling, using both intact bacterial infections and purified pathogen-associated molecular patterns such as lipid A and LPS (Khan et al., 2018b; Koppenol-Raab et al., 2017; Manes et al., 2017). Early studies by the Chen group combined subcellular fractionation with quantitative proteomics to examine Pam3CSK4- and LPS-stimulated mouse macrophages, enabling compartment-specific mapping of signaling responses during innate immune activation (Xue et al., 2008; Du et al., 2010). In a complementary approach, Lang et al. (Weintz et al., 2010) applied quantitative phosphoproteomics to define the temporal dynamics of TLR signaling, capturing both early and late phosphorylation events in LPS-stimulated macrophages.

Miller et al. further extended these approaches to host–pathogen systems by integrating proteomics and kinomics, including peptide array-based kinase activity profiling, to characterize signaling interactions between human macrophages and *S*. *aureus* (Miller et al., 2011). Collectively, these studies have provided detailed insights into protein abundance changes and phosphorylation-dependent signaling reprogramming during macrophage activation. Importantly, a global, unbiased quantitative phosphoproteomic analysis of macrophages following TLR activation identified 1,850 phosphoproteins and 6,956 phosphorylation sites, of which approximately two-thirds had not been previously described (Weintz et al., 2010). This work further demonstrated extensive rewiring of key signaling pathways, including PI3K/AKT and mTOR signaling, as well as activation of DNA damage response kinases such as ATM/ATR and modulation of cytoskeletal proteins, underscoring the broad scope of LPS-induced phosphorylation-mediated reprogramming (Weintz et al., 2010).

Decoding the intricate signaling networks induced by pathogenic stimuli remains a significant challenge in host-pathogen biology. Since macrophages often engage multiple TLRs concurrently during infection, it is essential to understand how distinct TLR pathways integrate and diverge under complex stimulation conditions. To address this, quantitative phosphoproteomics was used to map signaling dynamics downstream of TLR2, TLR4, and TLR7 in mouse macrophages stimulated with Pam3CSK4, LPS, and resiquimod over a 30-min time course (Sjoelund et al., 2014). This analysis revealed stimulus-specific regulation of proteins involved in immune signaling cascades, metabolic reprogramming, cytoskeletal organization, chromatin remodeling, and gene expression control.

Complementing these signaling studies, pulsed stable isotope labeling by amino acids in cell culture (pulsed SILAC) has enabled system-wide quantification of protein synthesis and degradation rates (Jovanovic et al., 2015). Jovanovic et al. (2015) integrated pulsed SILAC-based proteomics with transcriptomic profiling (RNA-seq) in LPS-stimulated mouse dendritic cells to capture dynamic immune responses over time. By combining these datasets, they constructed models of gene-specific transcription, translation, and protein turnover, revealing coordinated regulation across multiple layers of gene expression. This multi-omics approach further identified functionally related gene groups, including those associated with organelles, protein complexes, and immune pathways that are differentially controlled at the levels of protein production and degradation during inflammatory activation.

## 5 Preclinical and clinical status of HDTs

The antimicrobial functions of macrophages can be strengthened by cytokines, vitamins, and other regulatory molecules that activate intrinsic cellular defence pathways and enhance antimicrobial peptide (AMP) production. In TB, inflammatory pathways involving both pro- and anti-inflammatory mediators, particularly cytokines and lipid mediators, are dynamically and sequentially activated and suppressed during disease progression (Dorhoi and Kaufmann, 2014a). Current HDT strategies for TB include small molecules and biologics that modulate both pro- and anti-inflammatory pathways as adjuncts to conventional anti-TB treatment. *M. tuberculosis* alters arachidonic acid metabolism toward enhanced lipoxygenase (LOX) activity, leading to elevated production of the pro-necrotic lipid mediators lipoxin A4 (LXA4) and leukotriene B4 (LTB4). Therapeutic modulation of this pathway using zileuton, an approved 5-LOX inhibitor, or intranasal PGE2 administration has been reported to alleviate lung pathology in murine TB models (Mayer-Barber et al., 2014). The combined use of zileuton and PGE2 promotes protective IL-1β responses by reducing type I interferon signaling and restricting *M. tuberculosis* replication (Mayer-Barber et al., 2014). Nonsteroidal anti-inflammatory drugs (NSAIDs) such as Ibuprofen modulate inflammatory responses in TB by suppressing PGE2 production and enhancing lipoxin A4 (LXA4) generation, which contributes to both anti-inflammatory activity and regulation of *M. tuberculosis*-induced cell death. Through the reduction of prostaglandin and TNF levels, NSAIDs may help mitigate excessive local inflammation (Divangahi et al., 2013). Notably, aspirin and ibuprofen have progressed to clinical evaluation as adjunctive therapies for TB, including phase II trials in extensively drug-resistant (XDR) TB (Misra et al., 2010; Schoeman et al., 2011; Wallis et al., 2016; Fundació Institut Germans Trias i Pujol, 2016).

Given the close association between cell death and inflammatory amplification during *M. tuberculosis* infection, inhibition of TNF-induced necroptosis in infected macrophages has emerged as a potential host-directed therapeutic strategy. Agents such as alisporivir and desipramine restrict ROS-mediated necrotic cell death by inhibiting cyclophilin D and acid sphingomyelinase, respectively, thereby limiting the recruitment of Mtb-permissive cells and reducing bacterial dissemination (Roca and Ramakrishnan, 2013). Monoclonal antibodies targeting TNF, including Infliximab and Adalimumab, have demonstrated clinical benefit in some cases of severe TB (Blackmore et al., 2008; Wallis et al., 2009). In parallel, Etanercept, a soluble TNF receptor fusion protein, has exhibited modest adjunctive therapeutic effects (Wallis et al., 2009). Nevertheless, unlike etanercept, anti-TNF monoclonal antibodies can compromise granuloma integrity, thereby increasing the risk of latent TB reactivation (Dorhoi and Kaufmann, 2014b). Alternative approaches for modulating TNF-mediated inflammation include the use of thalidomide derivatives and phosphodiesterase (PDE) inhibitors (Ahidjo and Bishai, 2016). Thalidomide initially showed therapeutic promise in pulmonary TB (Tramontana et al., 1995), but its application was restricted by significant adverse events reported in pediatric TB trials, including hepatitis, severe neutropenia, thrombocytopenia, and skin reactions (Schoeman et al., 2004). Consequently, safer thalidomide analogues have been developed. Among these, PDE4 inhibitors that increase intracellular cAMP levels have shown efficacy as adjuncts to conventional anti-TB therapy in mouse and rabbit models (Koo et al., 2011; Subbian et al., 2016a; Subbian et al., 2016b). The nonspecific PDE inhibitor pentoxifylline was ineffective in TB clinical trials and, despite reducing TNF levels, worsened disease progression when used alone in murine models (Turner et al., 2001; Wallis et al., 1996). In contrast, the PDE inhibitor CC-11050 has completed phase I trials and is being evaluated as an adjunctive host-directed therapy alongside agents such as Everolimus, Auranofin, and Vitamin D to enhance standard TB chemotherapy (Wallis RS. et al., 2021; Wallis et al., 2004).

Sepsis is traditionally characterized by an uncontrolled host inflammatory response to invading pathogens. Host-directed anti-inflammatory therapies for viral infections and sepsis share overlapping strategies, with agents such as eritoran and S1P agonists targeting hyperinflammation and systemic immune dysregulation. However, only a few approaches, including COX-2 inhibitors, are being investigated for both TB and viral diseases, whereas glucocorticoid therapy is mainly under evaluation for TB.

Clinical translation of HDTs has advanced most prominently in TB, where several adjunctive immunomodulatory agents, including metformin, statins, vitamin D, phosphodiesterase inhibitors, corticosteroids, and mTOR-targeting compounds, have progressed to clinical trials with the aim of enhancing bacterial clearance while limiting excessive host inflammation. Although the outcomes of these studies have been variable, they have provided valuable insights into the feasibility of modulating host immune pathways as a complementary strategy to conventional antibiotic therapy. In contrast, the clinical development of HDTs for other MDR bacterial infections, including those caused by *S*. *aureus*, *P*. *aeruginosa*, *A*. *baumannii*, *K*. *pneumoniae*, and carbapenem-resistant Enterobacterales, remains relatively limited, with most therapeutic candidates still being evaluated in preclinical models or early-phase clinical studies. This discrepancy largely reflects the complexity of host–pathogen interactions, disease-specific immune responses, and the heterogeneous nature of bacterial infections, all of which present significant challenges for clinical translation.

A major obstacle to the successful implementation of HDTs is the lack of validated biomarkers that can reliably identify patients who are most likely to benefit from immune-modulating interventions. Since the immune response varies considerably depending on the pathogen, infection stage, disease severity, and host genetics, a “one-size-fits-all” therapeutic approach is unlikely to be effective. Consequently, biomarker-guided patient stratification and pharmacodynamics monitoring are expected to play critical roles in optimizing treatment efficacy, minimizing adverse effects, and determining the appropriate timing and duration of immunomodulatory therapy. In addition, standardized biomarkers are needed to monitor therapeutic responses and evaluate treatment outcomes in clinical trials. Despite encouraging progress, several challenges continue to impede the clinical translation of HDTs, including limited long-term safety data, uncertainties regarding optimal dosing and treatment duration, regulatory hurdles for immune-modulating agents, and the absence of standardized clinical endpoints across trials. Moreover, the complexity of immune modulation necessitates careful balancing of antimicrobial immunity with the prevention of immunopathology, as excessive immune activation or suppression may adversely affect patient outcomes. Therefore, future research should prioritize well-designed multicenter clinical trials, standardized biomarker validation, and the integration of precision medicine frameworks to accelerate the development of safe, effective, and pathogen-specific host-directed therapeutic strategies for combating bacterial infections, particularly those caused by antimicrobial-resistant pathogens.

## 6 Limitations and challenges

A personalized medicine approach may be essential for recalibrating host immune responses, as different pathogens are likely to require varying degrees and mechanisms of immunomodulation. For instance, *S. aureus* infection is known to inhibit apoptosis-mediated killing in macrophages, potentially necessitating stronger immune enhancement or alternative therapeutic interventions. Conversely, in *S. pneumoniae* infection, where apoptosis-associated bacterial clearance is already activated, only a moderate level of immune adjustment may be required in certain individuals (Preston et al., 2019). Another important limitation of immune recalibration strategies is the need to avoid excessive amplification of host responses, as uncontrolled production of microbicidal mediators or inflammatory cells may contribute to tissue injury and immunopathological damage (Han and Mallampalli, 2015). The risk of such detrimental effects is expected to be minimized when the induced responses are localized, transient, intracellular, and concentrated near the site of bacterial infection. Consequently, successful therapeutic modulation will depend on accurate assessment of patient-specific immune responses using reliable biomarkers and advanced imaging techniques (Robles-Medranda, 2016).

Following successful *in vivo* validation of a host-directed therapeutic hypothesis, the subsequent translation of these findings into human clinical settings remains a substantial challenge. In contrast to conventional antibiotics, which typically demonstrate relatively predictable pharmacokinetic and pharmacodynamic profiles, host-directed therapeutics exert their effects indirectly through modulation of host cellular mechanisms and immune pathways. Consequently, therapeutic outcomes are influenced not only by drug exposure but also by complex downstream immunological events within the host. This multifaceted mode of action increases the difficulty of interpreting efficacy data obtained from animal models and introduces considerable uncertainty during translation to humans. Moreover, interspecies differences in immune architecture, variability in host–pathogen interactions, and heterogeneity in individual immune responses further complicate the clinical development and evaluation of host-directed therapeutic strategies.

In cases of severe or life-threatening infections, withholding standard antibiotic therapy from patients is ethically unacceptable, making it necessary to evaluate host-directed therapeutics primarily in combination with conventional antibiotics rather than as standalone treatments. Consequently, clinical trials investigating such combination therapies must demonstrate a clear therapeutic benefit over the existing standard of care, which typically consists of antibiotic treatment alone. Achieving statistically significant improvements under these conditions can be particularly challenging due to the already established efficacy of standard antimicrobial regimens. Furthermore, HDTs encompass a broad and heterogeneous range of drug classes, each possessing distinct mechanisms of action, pharmacological profiles, and safety considerations. Therefore, careful assessment of potential risks, adverse immunological effects, and drug-specific concerns is essential during the clinical development and evaluation of these therapeutic strategies.

A significant concern associated with HDT strategies is that many of the proposed molecular targets are integrated into multiple physiological and immunological signaling networks, thereby increasing the likelihood of unintended systemic effects and toxicity. Therapeutic agents designed to enhance immune activity may inadvertently trigger uncontrolled inflammatory responses, leading to severe complications such as cytokine storm or systemic inflammatory response syndrome, both of which can impair host defense mechanisms and worsen disease outcomes. Furthermore, the success of host-directed interventions is closely linked to the timing of administration, as these therapies generally act on distinct stages of infection and immune activation.

Another significant barrier is the lack of robust predictive biomarkers capable of stratifying patients according to their immune status and identifying those most likely to benefit from specific immunomodulatory interventions. The host immune response to bacterial infection is highly heterogeneous and is influenced by multiple factors, including age, genetic background, underlying comorbidities, microbiome composition, pathogen virulence, infection site, and disease severity. Consequently, therapeutic strategies that are effective in one patient population may show limited efficacy or even detrimental effects in another, highlighting the need for precision medicine approaches that integrate immunophenotyping and biomarker-guided patient selection.

Optimization of the dosage and timing of HDTs is essential to achieve an appropriate balance between therapeutic benefit and safety, with particular emphasis on minimizing treatment-associated adverse effects. The host immune response evolves dynamically throughout the course of infection, transitioning from an initial antimicrobial inflammatory phase to a resolution and tissue repair phase. Immunostimulatory interventions administered during early infection may enhance pathogen clearance; however, prolonged or excessive immune activation can exacerbate tissue damage, systemic inflammation, and organ dysfunction. Conversely, anti-inflammatory therapies may reduce immunopathology but could impair bacterial clearance if administered prematurely or inappropriately. Therefore, understanding the temporal dynamics of immune responses and defining therapeutic windows remain essential prerequisites for the successful clinical application of HDTs (Netea et al., 2020). TLR2 is considered a promising therapeutic target for the treatment of infectious diseases, inflammatory disorders, and allergic conditions due to its central role in innate immune signaling. However, persistent or uncontrolled activation of TLR2 can promote prolonged inflammatory responses, potentially contributing to the development and progression of autoimmune diseases and chronic inflammatory conditions (Gorecki et al., 2021). Similarly, extensive investigations have explored phosphodiesterase-4 inhibition as a promising therapeutic approach for a broad spectrum of human diseases. Nevertheless, the clinical advancement of many PDE4 inhibitors has been significantly constrained by their limited therapeutic index and the prevalence of considerable adverse effects observed during clinical evaluation.

Patient heterogeneity further complicates the clinical development of HDTs. Individuals with diabetes, cancer, autoimmune diseases, chronic inflammatory disorders, or those receiving immunosuppressive therapy often exhibit markedly altered immune responses compared with otherwise healthy individuals. Likewise, elderly patients frequently experience immunosenescence, whereas pediatric patients possess distinct immune developmental profiles. These variations may significantly influence treatment outcomes and necessitate individualized therapeutic strategies rather than a universal approach. Future clinical trials should therefore incorporate immune profiling and stratified study designs to account for patient-specific biological variability.

Regulatory considerations also represent an important obstacle to HDT development. Unlike conventional antibiotics that directly target pathogens, HDTs modulate host immune pathways and therefore require comprehensive evaluation of both efficacy and long-term safety. Potential risks include immune dysregulation, excessive inflammation, autoimmune manifestations, opportunistic infections, and interference with protective immune memory. Consequently, regulatory agencies require extensive safety assessment, long-term follow-up, and carefully designed clinical endpoints before approval of immunomodulatory therapies. The absence of standardized biomarkers and validated surrogate endpoints further complicates regulatory evaluation and prolongs clinical development. Economic and logistical challenges must also be considered before widespread implementation of HDTs. Many emerging strategies rely on advanced molecular diagnostics, high-throughput multi-omics technologies, and personalized biomarker analyses, all of which substantially increase healthcare costs and infrastructure requirements. These limitations may restrict accessibility in low- and middle-income countries, where the burden of antimicrobial resistance is greatest. Developing cost-effective diagnostic platforms, standardized laboratory workflows, and scalable precision medicine approaches will therefore be essential for ensuring equitable clinical implementation worldwide.

A major challenge underlying these toxicities is the inadequate selectivity of these compounds across distinct PDE4 subfamilies, which has been associated with undesirable side effects, particularly emesis and other gastrointestinal disturbances. Consequently, these safety concerns have led to the discontinuation of several PDE4 inhibitors during clinical development and have limited their broader therapeutic application (Li et al., 2023). Moreover, HDTs may produce unintended off-target effects that interfere with normal cellular and physiological processes, thereby raising concerns regarding safety and therapeutic specificity. In addition, inter-individual differences such as genetic variability, immune status, and underlying health conditions can significantly influence host responses to treatment, resulting in variable therapeutic efficacy across patient populations. Collectively, these factors present major challenges to the development of broadly applicable and universally effective HDT strategies (Liu X. et al., 2022). Therefore, precise characterization of the therapeutic window is critical to ensure that immune modulation occurs at an appropriate stage of disease progression, thereby optimizing clinical benefit while reducing the risk of excessive inflammation and treatment-associated toxicity.

High-throughput approaches such as yeast two-hybrid (Y2H), RNA interference (RNAi), and siRNA screening have greatly expanded the identification of host factors involved in bacterial infection; however, their interpretation is limited by reproducibility issues and assay-specific artifacts. Y2H-based interaction mapping can generate false-positive interactions due to non-physiological expression conditions, protein misfolding, or reporter-system artifacts, whereas RNAi/siRNA screens are affected by incomplete knockdown, off-target effects, and variable silencing efficiency (Venkatesan et al., 2009; Echeverri et al., 2006; Jackson and Linsley, 2010; Johnson et al., 2007). Therefore, candidate host targets identified from these platforms require validation using orthogonal experimental approaches. In addition, integration of multi-omics datasets is challenged by batch effects arising from differences in sample processing, experimental platforms, and computational pipelines. Effective normalization, batch correction, and cross-platform harmonization strategies are essential to distinguish true biological signals from technical variability and improve the reliability of systems-level host target identification.

Cell-line-derived eQTL and phosphoproteomic studies provide valuable insights into host regulatory networks during infection; however, their translation to human disease is limited by differences in cellular context, tissue specificity, immune heterogeneity, and experimental conditions (Fairfax). Genetic regulatory effects identified in immortalized cell models may not accurately reflect host responses during natural infection, while phosphoproteomic alterations can vary depending on infection stage and host–pathogen interactions. Therefore, integration with patient-derived datasets, clinical cohorts, and orthogonal functional validation is essential to identify robust host targets for therapeutic development.

## 7 Conclusion

In recent years, advances in the characterization of immune function pathways have provided some of the most promising opportunities for the development of HDTs. HDTs, which act by modulating host cell processes, boosting immune defenses, and controlling excessive inflammation, offer significant potential for the targeted and symptomatic treatment of infectious diseases. By maintaining a balanced host response at the site of infection, these therapies can improve disease outcomes. Throughout the progression of disease, multiple immune-related pathways may serve as potential therapeutic targets, with HDTs typically designed to act at specific stages of infection. The ultimate effect of such interventions depends on whether a given pathway is inhibited or activated, as the use of receptor-specific agonists or antagonists can markedly influence immune responses and, consequently, disease outcomes. To minimize the risk of unintentionally disrupting protective immune mechanisms, immune-profiling diagnostics would likely be necessary to accurately determine and characterize a patient’s stage of infection. However, these time-dependent HDT strategies remain limited, as they lack the flexibility required to effectively manage disease across the full course of infection. Moreover, despite extensive identification of key host pathways and proteins, a substantial gap remains between initial hypotheses and preclinical validation, mainly because of the lack of appropriate tool compounds for evaluation in animal infection models.

## References

[B1] Aebersold R. Mann M. (2016). Mass-spectrometric exploration of proteome structure and function. Nature 537 (7620), 347–355. 10.1038/nature19949 27629641

[B2] Ahidjo B. A. Bishai W. R. (2016). Phosphodiesterase inhibitors as adjunctive therapies for tuberculosis. EBioMedicine 4 (7), 8. 10.1016/j.ebiom.2016.02.016 26981560 PMC4776226

[B3] Albers H. M. Kuijl C. Bakker J. Hendrickx L. Wekker S. Farhou N. (2014). Integrating chemical and genetic silencing strategies to identify host kinase-phosphatase inhibitor networks that control bacterial infection. ACS Chem. Biol. 9, 414–422. 10.1021/cb400421a 24274083 PMC3934374

[B4] Andersson J. A. Sha J. Kirtley M. L. Reyes E. Fitts E. C. Dann S. M. (2017). Combating multidrug-resistant pathogens with host-directed nonantibiotic therapeutics. Antimicrob. Agents Chemother. 62 (1), e01943. 10.1128/AAC.01943-17 29109161 PMC5740341

[B5] Bagnoli F. Fontana M. R. Soldaini E. Mishra R. P. Fiaschi L. Cartocci E. (2015). Vaccine composition formulated with a novel TLR7-dependent adjuvant induces high and broad protection against *Staphylococcus aureus* . Proc. Natl. Acad. Sci. U S A. 112, 3680–3685. 10.1073/pnas.1424924112 25775551 PMC4378396

[B6] Bakkeren E. Huisman J. S. Fattinger S. A. Hausmann A. Furter M. Egli A. (2019). Salmonella persisters promote the spread of antibiotic resistance plasmids in the gut. Nature 573, 276–280. 10.1038/s41586-019-1521-8 31485077 PMC6744281

[B7] Banerjee S. K. Thurlow L. R. Kannan K. Richardson A. R. (2024). Glucose transporter 1 is essential for the resolution of methicillin-resistant *S. aureus* skin and soft tissue infections. Cell Rep. 43 (7), 114486. 10.1016/j.celrep.2024.114486 PMC11323221 38990718

[B8] Barrett T. C. Mok W. W. K. Murawski A. M. Brynildsen M. P. (2019). Enhanced antibiotic resistance development from fluoroquinolone persisters after a single exposure to antibiotic. Nat. Commun. 10, 1177. 10.1038/s41467-019-09058-4 30862812 PMC6414640

[B9] Bhatt K. Bhagavathula M. Verma S. Timmins G. S. Deretic V. P. Ellner J. J. (2021). Rapamycin modulates pulmonary pathology in a murine model of *Mycobacterium tuberculosis* infection. Dis. Model Mech. 14, dmm049018. 10.1242/dmm.049018 34486033 PMC8560501

[B10] Blackmore T. K. Manning L. Taylor W. J. Wallis R. S. (2008). Therapeutic use of infliximab in tuberculosis to control severe paradoxical reaction of the brain and lymph nodes. Clin. Infect. Dis. 47, E83–E85. 10.1086/592695 18840076

[B11] Boersema P. J. Kahraman A. Picotti P. (2015). Proteomics beyond large-scale protein expression analysis. Curr. Opin. Biotechnol. 34, 162–170. 10.1016/j.copbio.2015.01.005 25636126

[B12] Bouzeyen R. Haoues M. Barbouche M.-R. Singh R. Essafi M. (2019). FOXO3 transcription factor regulates IL-10 expression in mycobacteria-infected macrophages, tuning their polarization and the subsequent adaptive immune response. Front. Immunol. 10, 2922. 10.3389/fimmu.2019.02922 31921181 PMC6927284

[B13] Bravo-Santano N. Ellis J. K. Mateos L. M. Calle Y. Keun H. C. Behrends V. (2018). Intracellular *Staphylococcus aureus* infection modulates host central carbon metabolism to activate autophagy. mSphere 3 (4), e00374. 10.1128/mSphere.00374-18 30089650 PMC6083095

[B14] Bruns H. Stegelmann F. Fabri M. Döhner K. van Zandbergen G. Wagner M. (2012). Abelson tyrosine kinase controls phagosomal acidification required for killing of *Mycobacterium tuberculosis* in human macrophages. J. Immunol. 189, 4069–4078. 10.4049/jimmunol.1201538 22988030 PMC3684563

[B15] Cassini A. Högberg L. D. Plachouras D. Quattrocchi A. Hoxha A. Simonsen G. S. (2019). Attributable deaths and disability-adjusted life-years caused by infections with antibiotic-resistant bacteria in the EU and the european economic area in 2015: a population-level modelling analysis. Lancet Infect. Dis. 19 (1), 56–66. 10.1016/s1473-3099(18)30605-4 PMC6300481 30409683

[B16] Casulli J. Fife M. E. Houston S. A. Rossi S. Dow J. Williamson E. D. (2019). CD200R deletion promotes A neutrophil niche for Francisella tularensis and increases infectious burden and mortality. Nat. Commun. 10 (1), 2121. 10.1038/s41467-019-10156-6 31073183 PMC6509168

[B17] Cavallo I. Sivori F. Mastrofrancesco A. Abril E. Pontone M. Di Domenico E. G. (2024). Bacterial biofilm in chronic wounds and possible therapeutic approaches. Biology 13 (2), 109. 10.3390/biology13020109 PMC10886835 38392327

[B18] Ceballos-Olvera I. Sahoo M. Miller M. A. Del Barrio L. Re F. (2011). Inflammasome-dependent pyroptosis and IL-18 protect against burkholderia pseudomallei lung infection while IL-1beta is deleterious. PLoS Pathog. 7, e1002452. 10.1371/journal.ppat.1002452 22241982 PMC3248555

[B19] Centanni D. Henricks P. A. J. Engels F. (2023). The therapeutic potential of resolvins in pulmonary diseases. Eur. J. Pharmacol. 958, 176047. 10.1016/j.ejphar.2023.176047 37742814

[B20] Chang E. Cavallo K. Behar S. M. (2025). CD4 T cell dysfunction is associated with bacterial recrudescence during chronic tuberculosis. Nat. Commun. 16 (1), 2636. 10.1038/s41467-025-57819-1 PMC11914476 40097414

[B21] Charoensup J. Sermswan R. W. Paeyao A. Promakhejohn S. Punasee S. Chularari C. (2014). High HMGB1 level is associated with poor outcome of septicemic melioidosis. Int. J. Infect. Dis. 28, 111–116. 10.1016/j.ijid.2014.07.025 25263503

[B22] Chatterjee S. Yabaji S. M. Rukhlenko O. S. Bhattacharya B. Waligurski E. Vallavoju N. (2021). Channeling macrophage polarization by rocaglates increases macrophage resistance to *Mycobacterium tuberculosis* . iScience 24, 102845. 10.1016/j.isci.2021.102845 PMC8333345 34381970

[B23] Chen W. (2020). Host-pathogen interactions in *Acinetobacter baumannii* infection: recent advances and future challenges. Future Microbiol. 15, 841–845. 10.2217/fmb-2020-0032 32657617

[B24] Chen F. Wang N. Liao J. Jin M. Qu F. Wang C. (2024). Esculetin rebalances M1/M2 macrophage polarization to treat sepsis-induced acute lung injury through regulating metabolic reprogramming. J. Cell Mol. Med. 28 (21), e70178. 10.1111/jcmm.70178 PMC11558263 39535339

[B25] Chiang C. Y. Uzoma I. Lane D. J. Memiševic´ V. Alem F. Yao K. (2015). A reverse-phase protein microarray-based screen identifies host signaling dynamics upon burkholderia spp. infection. Front. Microbiol. 6, 683. 10.3389/fmicb.2015.00683 26284031 PMC4515560

[B26] Chiu H. C. Soni S. Kulp S. K. Curry H. Wang D. Gunn J. S. (2009a). Eradication of intracellular Francisella tularensis in THP-1 human macrophages with a novel autophagy inducing agent. J. Biomed. Sci. 16, 110. 10.1186/1423-0127-16-110 20003180 PMC2801672

[B27] Chiu H. C. Kulp S. K. Soni S. Wang D. Gunn J. S. Schlesinger L. S. (2009b). Eradication of intracellular *Salmonella enterica* serovar typhimurium with a small-molecule, host cell-directed agent. Antimicrob. Agents Chemother. 53, 5236–5244. 10.1128/AAC.00555-09 19805568 PMC2786354

[B28] Choi S. W. Gu Y. Peters R. S. Salgame P. Ellner J. J. Timmins G. S. (2018). Ambroxol induces autophagy and potentiates rifampin antimycobacterial activity. Antimicrob. Agents Chemother. 62, e01019-18. 10.1128/AAC.01019-18 30012752 PMC6125555

[B29] Coll R. C. Robertson A. A. Chae J. J. Higgins S. C. Muñoz-Planillo R. Inserra M. C. (2015). A small-molecule inhibitor of the NLRP3 inflammasome for the treatment of inflammatory diseases. Nat. Med. 21, 248–255. 10.1038/nm.3806 25686105 PMC4392179

[B30] Critchley J. A. Young F. Orton L. Garner P. (2013). Corticosteroids for prevention of mortality in people with tuberculosis: a systematic review and meta-analysis. Lancet Infect. Dis. 13, 223–237. 10.1016/S1473-3099(12)70321-3 23369413

[B31] Cullinane M. Gong L. Li X. Lazar-Adler N. Tra T. Wolvetang E. (2008). Stimulation of autophagy suppresses the intracellular survival of burkholderia pseudomallei in mammalian cell lines. Autophagy 4, 744–753. 10.4161/auto.6246 18483470

[B32] Dalli J. Chiang N. Serhan C. N. (2015). Elucidation of novel 13-series resolvins that increase with atorvastatin and clear infections. Nat. Med. 21, 1071–1075. 10.1038/nm.3911 26236990 PMC4560998

[B33] Deretic V. (2012). Autophagy as an innate immunity paradigm: expanding the scope and repertoire of pattern recognition receptors. Curr. Opin. Immunol. 24, 21–31. 10.1016/j.coi.2011.10.006 22118953 PMC3288884

[B34] Divangahi M. Behar S. M. Remold H. (2013). in New Paradigm of Immunity to Tuberculosis. Editor Divangahi M. (Springer), 103–120.

[B35] Dorhoi A. Kaufmann S. H. E. (2014a). Perspectives on host adaptation in response to mycobacterium tuberculosis: modulation of inflammation. Sem. Immunol 26, 533–542. 10.1016/j.smim.2014.10.002 25453228

[B36] Dorhoi A. Kaufmann S. H. E. (2014b). Tumor necrosis factor-α in mycobacterial infection. Semin. Immunol. 26, 203–209. 10.1016/j.smim.2014.04.003 24819298

[B37] Du R. Long J. Yao J. Dong Y. Yang X. Tang S. (2010). Subcellular quantitative proteomics reveals multiple pathway cross-talk that coordinates specific signaling and transcriptional regulation for the early host response to LPS. J. Proteome Res. 9 (4), 1805–1821. 10.1021/pr900962c 20158268

[B38] Dumigan A. Cappa O. Morris B. Sá Pessoa J. Calderon-Gonzalez R. Mills G. (2022). *In vivo* single-cell transcriptomics reveal *Klebsiella pneumoniae* skews lung macrophages to promote infection. EMBO Mol. Med. 14 (12), e16888. 10.15252/emmm.202216888 PMC9727930 36337046

[B39] Dutta N. K. Bruiners N. Zimmerman M. D. Tan S. Dartois V. Gennaro M. L. (2020). Adjunctive host-directed therapy with statins improves tuberculosis-related outcomes in mice. J. Infect. Dis. 221, 1079–1087. 10.1093/infdis/jiz517 31605489 PMC7325721

[B40] D’elia R. V. Laws T. R. Carter A. Lukaszewski R. Clark G. C. (2013). Targeting the “Rising damp” during A Francisella tularensis infection. Antimicrob. Agents Chemother. 57, 4222–4228. 10.1128/AAC.01885-12 PMC3754292 23796927

[B41] Echeverri C. J. Beachy P. A. Baum B. Boutros M. Buchholz F. Chanda S. K. (2006). Minimizing the risk of reporting false positives in large-scale RNAi screens. Nat. Methods 3 (10), 777–779. 10.1038/nmeth1006-777 16990807

[B42] Fairfax B. P. Humburg P. Makino S. Naranbhai V. Wong D. Lau E. (2014). Innate immune activity conditions the effect of regulatory variants upon monocyte gene expression. Science 343 (6175), 1246949. 10.1126/science.1246949 24604202 PMC4064786

[B43] Fisher R. A. Gollan B. Helaine S. (2017). Persistent bacterial infections and persister cells. Nat. Rev. Microbiol. 15, 453–464. 10.1038/nrmicro.2017.42 28529326

[B44] Frank C. G. Reguerio V. Rother M. Moranta D. Maeurer A. P. Garmendia J. (2013). *Klebsiella pneumoniae* targets an EGF receptor-dependent pathway to subvert inflammation. Cell Microbiol. 15, 1212–1233. 10.1111/cmi.12110 23347154

[B45] Frank D. J. Horne D. J. Dutta N. K. Shaku M. T. Madensein R. Hawn T. R. (2019). Remembering the host in tuberculosis drug development. J. Infect. Dis. 219, 1518–1524. 10.1093/infdis/jiy712 30590592 PMC6562157

[B46] Fundació Institut Germans Trias i Pujol (2016). Potential efficacy and safety of using adjunctive ibuprofen for XDR-TB tuberculosis (NSAIDS-XDRTB) (clinicaltrials.Gov identifier NCT02781909). Available online at: https://clinicaltrials.gov/ct2/show/NCT02781909.

[B47] Garber M. Yosef N. Goren A. Raychowdhury R. Thielke A. Guttman M. (2012). A high-throughput chromatin immunoprecipitation approach reveals principles of dynamic gene regulation in mammals. Mol. Cell 47 (5), 810–822. 10.1016/j.molcel.2012.07.030 22940246 PMC3873101

[B48] Garcia Jacobo R. E. Serrano C. J. Enciso Moreno J. A. Gaspar Ramírez O. Trujillo Ochoa J. L. Uresti Rivera E. E. (2014). Analysis of Th1, Th17 and regulatory T cells in tuberculosis case contacts. Cell Immunol. 289, 167–173. 10.1016/j.cellimm.2014.03.010 24841855

[B49] GBD 2021 Antimicrobial Resistance Collaborators (2024). Global burden of bacterial antimicrobial resistance 1990-2021: a systematic analysis with forecasts to 2050. Lancet 404 (10459), 1199–1226. 10.1016/S0140-6736(24)01867-1 39299261 PMC11718157

[B50] Ge M. Ruan Z. Zhu Y. X. Wu W. Yang C. Lin H. (2024). A natural killer cell mimic against intracellular pathogen infections. Sci. Adv. 10 (44), eadp3976. 10.1126/sciadv.adp3976 39475620 PMC11524181

[B51] Gholap A. D. Khuspe P. R. Pardeshi S. R. Uddin M. J. Das U. Hatvate N. T. (2025). Achieving optimal health with host-directed therapies (HDTs) in infectious diseases—A new horizon. Adv. Ther. 8, 2400169. 10.1002/adtp.202400169

[B52] Gorecki A. M. Anyaegbu C. C. Anderton R. S. (2021). TLR2 andTLR4inParkinson’sdiseasepathogenesis: the environment takes a toll on the gut. Transl. Neurodegener. 10 (1), 47. 10.1186/s40035-021-00271-0 PMC8609261 34814947

[B53] Gupta A. Sharma D. Meena J. Pandya S. Sachan M. Kumar S. (2016). Preparation and preclinical evaluation of inhalable particles containing rapamycin and anti-tuberculosis agents for induction of autophagy. Pharm. Res. 33, 1899–1912. 10.1007/s11095-016-1926-0 27095353

[B54] Ha K. P. Edwards A. M. (2021). DNA repair in *Staphylococcus aureus* . Microbiol. Mol. Biol. Rev. 85 (4), e0009121. 10.1128/mmbr.00091-21 34523959 PMC8483670

[B55] Han S. Mallampalli R. K. (2015). The acute respiratory distress syndrome: from mechanism to translation. J. Immunol. 194, 855–860. 10.4049/jimmunol.1402513 25596299 PMC4299926

[B56] Harrison A. Dubois L. G. St John-Williams L. Moseley M. A. Hardison R. L. Heimlich D. R. (2016). Comprehensive proteomic and metabolomic signatures of nontypeable haemophilus influenzae-induced acute otitis media reveal bacterial aerobic respiration in an immunosuppressed environment. Mol. Cell Proteomics 15, 1117–1138. 10.1074/mcp.M115.052498 26711468 PMC4813693

[B57] Heemskerk M. T. Korbee C. J. Esselink J. J. Dos Santos C. C. van Veen S. Gordijn I. F. (2021). Repurposing diphenylbutylpiperidine-class antipsychotic drugs for host-directed therapy of *Mycobacterium tuberculosis* and *Salmonella enterica* infections. Sci. Rep. 11, 19634. 10.1038/s41598-021-98980-z PMC8490354 34608194

[B58] Higgins D. F. Kimura K. Bernhardt W. M. Shrimanker N. Akai Y. Hohenstein B. (2007). Hypoxia promotes fibrogenesis *in vivo via* HIF-1 stimulation of epithelial-to-mesenchymal transition. J. Clin. Investigation 117 (12), 3810–3820. 10.1172/JCI30487 18037992 PMC2082142

[B59] Hirano T. Kodama S. Kawano T. Maeda K. Suzuki M. (2011). Monophosphoryl lipid A induced innate immune responses *via* TLR4 to enhance clearance of nontypeable Haemophilus influenzae and Moraxella catarrhalis from the nasopharynx in mice. FEMS Immunol. Med. Microbiol. 63, 407–417. 10.1111/j.1574-695X.2011.00866.x 22092567

[B60] Hoogendijk A. J. Roelofs J. J. Duitman J. van Lieshout M. H. Blok D. C. van der Poll T. (2012). R-roscovitine reduces lung inflammation induced by lipoteichoic acid and Streptococcus pneumoniae. Mol. Med. 18 (1), 1086–1095. 10.2119/molmed.2012.00033 22692577 PMC3475337

[B61] Huang J. Brumell J. H. (2014). Bacteria-autophagy interplay: a battle for survival. Nat. Rev. Microbiol. 12, 101–114. 10.1038/nrmicro3160 24384599 PMC7097477

[B62] Huang L. Nazarova E. V. Tan S. Liu Y. Russell D. G. (2018). Growth of *Mycobacterium tuberculosis in vivo* segregates with host macrophage metabolism and ontogeny. J. Exp. Med. 215, 1135–1152. 10.1084/jem.20172020 29500179 PMC5881470

[B63] Imtiyaz H. Z. Simon M. C. (2010). Hypoxia-inducible factors as essential regulators of inflammation. Curr. Top. Microbiol. Immunol. 345, 105–120. 10.1007/82_2010_74 20517715 PMC3144567

[B64] Jackson A. L. Linsley P. S. (2010). Recognizing and avoiding siRNA off-target effects for target identification and therapeutic application. Nat. Rev. Drug Discov. 9 (1), 57–67. 10.1038/nrd3010 20043028

[B65] Jenum S. Tonby K. Rueegg C. S. Rühwald M. Kristiansen M. P. Bang P. (2021). A phase I/II randomized trial of H56:IC31 vaccination and adjunctive cyclooxygenase-2-inhibitor treatment in tuberculosis patients. Nat. Commun. 12 (1), 6774. 10.1038/s41467-021-27029-6 PMC8608791 34811370

[B66] Jhilta A. Jadhav K. Singh R. Ray E. Kumar A. Singh A. K. (2024). Breaking the cycle: matrix metalloproteinase inhibitors as an alternative approach in managing tuberculosis pathogenesis and progression. ACS Infect. Dis. 10 (8), 2567–2583. 10.1021/acsinfecdis.4c00385 39038212

[B67] Johnson W. E. Li C. Rabinovic A. (2007). Adjusting batch effects in microarray expression data using empirical bayes methods. Biostatistics 8 (1), 118–127. 10.1093/biostatistics/kxj037 16632515

[B68] Jordan P. M. Gerstmeier J. Pace S. Bilancia R. Rao Z. Börner F. (2020). Staphylococcus aureus-derived α-hemolysin evokes generation of specialized pro-resolving mediators promoting inflammation resolution. Cell Rep. 33 (2), 108247. 10.1016/j.celrep.2020.108247 33053344 PMC7729929

[B69] Jorge J. H. Graciela C. Pablo A. P. Luis S. H. J. (2012). A life-threatening central nervous system-tuberculosis inflammatory reaction nonresponsive to corticosteroids and successfully controlled by infliximab in a young patient with a variant of juvenile idiopathic arthritis. J. Clin. Rheumatol. 18, 189–191. 10.1097/RHU.0b013e318258b725 22647865

[B70] Jovanovic M. Rooney M. S. Mertins P. Przybylski D. Chevrier N. Satija R. (2015). Immunogenetics. Dynamic profiling of the protein life cycle in response to pathogens. Science 347 (6226), 1259038. 10.1126/science.1259038 25745177 PMC4506746

[B71] Kale S. D. Dikshit N. Kumar P. Balamuralidhar V. Khameneh H. J. Bin Abdul Malik N. (2017). Nod2 is required for the early innate immune clearance of *Acinetobacter baumannii* from the lungs. Sci. Rep. 7 (1), 17429. 10.1038/s41598-017-17653-y 29234083 PMC5727160

[B72] Kaufmann S. H. E. Dorhoi A. Hotchkiss R. S. Bartenschlager R. (2018). Host-directed therapies for bacterial and viral infections. Nat. Rev. Drug Disc 17, 35–56. 10.1038/nrd.2017.162 28935918 PMC7097079

[B73] Kewcharoenwong C. Rinchai D. Utispan K. Suwannasaen D. Bancroft G. J. Ato M. (2013). Glibenclamide reduces proinflammatory cytokine production by neutrophils of diabetes patients in response to bacterial infection. Sci. Rep. 3, 3363. 10.1038/srep03363 24285369 PMC3842541

[B74] Khan M. M. Ernst O. Sun J. Fraser I. D. C. Ernst R. K. Goodlett D. R. (2018a). Mass spectrometry-based structural analysis and systems immunoproteomics strategies for deciphering the host response to endotoxin. J. Mol. Biol. 430 (17), 2641–2660. 10.1016/j.jmb.2018.06.032 29949751

[B75] Khan M. M. Koppenol-Raab M. Kuriakose M. Manes N. P. Goodlett D. R. Nita-Lazar A. (2018b). Host-pathogen dynamics through targeted secretome analysis of stimulated macrophages. J. Proteomics 189, 34–38. 10.1016/j.jprot.2018.03.016 29572161 PMC6149218

[B76] Kierbel A. Gassama-Diagne A. Rocha C. Radoshevich L. Olson J. Mostov K. (2007). *Pseudomonas aeruginosa* exploits a PIP3-dependent pathway to transform apical into basolateral membrane. J. Cell Biol. 177, 21–27. 10.1083/jcb.200605142 17403925 PMC2064102

[B77] Kim L. (2007). Persister cells, dormancy and infectious disease. Nat. Rev. Microbiol. 5, 48–56. 10.1038/nrmicro1557 17143318

[B78] Kim L. (2010). Persister cells. Annu. Rev. Microbiol. 64, 357–372. 10.1146/annurev.micro.112408.134306 20528688

[B79] Kim S. Becker J. Bechheim M. Kaiser V. Noursadeghi M. Fricker N. (2014). Characterizing the genetic basis of innate immune response in TLR4-Activated human monocytes. Nat. Commun. 5, 5236. 10.1038/ncomms6236 25327457

[B80] Koh G. C. Maude R. R. Schreiber M. F. Limmathurotsakul D. Wiersinga W. J. Wuthiekanun V. (2011). Glyburide is anti-inflammatory and associated with reduced mortality in melioidosis. Clin. Infect. Dis. 52, 717–725. 10.1093/cid/ciq192 21293047 PMC3049341

[B81] Koh G. C. Weehuizen T. A. Breitbach K. Krause K. de Jong H. K. Kager L. M. (2013). Glyburide reduces bacterial dissemination in a mouse model of melioidosis. PLoS Negl. Trop. Dis. 7, e2500. 10.1371/journal.pntd.0002500 24147174 PMC3798430

[B82] Koo M. S. Manca C. Yang G. O'Brien P. Sung N. Tsenova L. (2011). Phosphodiesterase 4 inhibition reduces innate immunity and improves isoniazid clearance of *Mycobacterium tuberculosis* in the lungs of infected mice. PLoS ONE 6, e17091. 10.1371/journal.pone.0017091 21364878 PMC3045423

[B83] Koppenol-Raab M. Sjoelund V. Manes N. P. Gottschalk R. A. Dutta B. Benet Z. L. (2017). Proteome and secretome analysis reveals differential post-transcriptional regulation of toll-like receptor responses. Mol. Cell. Proteomics 16 (4 Suppl. 1), S172–S186. 10.1074/mcp.M116.064261 28235783 PMC5393387

[B84] Krachler A. M. Woolery A. R. Orth K. (2011). Manipulation of kinase signalling by bacterial pathogens. J. Cell Biol. 195, 1083–1092. 10.1083/jcb.201107132 22123833 PMC3246894

[B85] Kramer L. Turk D. Turk B. (2017). The future of cysteine cathepsins in disease management. Trends Pharmacol. Sci. 38, 873–898. 10.1016/j.tips.2017.06.003 28668224

[B86] Kuhns D. B. Alvord W. G. Heller T. Feld J. J. Pike K. M. Marciano B. E. (2010). Residual NADPH oxidase and survival in chronic granulomatous disease. N. Engl. J. Med. 363, 2600–2610. 10.1056/NEJMoa1007097 21190454 PMC3069846

[B87] Kuijl C. Savage N. D. Marsman M. Tuin A. W. Janssen L. Egan D. A. (2007). Intracellular bacterial growth is controlled by a kinase network around PKB/AKT1. Nature 450, 725–730. 10.1038/nature06345 18046412

[B88] Kumari A. Pahuja I. Negi K. Ghoshal A. Mukopadhyay S. Agarwal M. (2023). Withaferin a protects against primary and recurrent tuberculosis by modulating Mycobacterium-specific host immune responses. Microbiol. Spectr. 11, e0058323. 10.1128/spectrum.00583-23 PMC10100980 36916966

[B89] Lai S. C. Devenish R. J. (2012). LC3-associated phagocytosis (LAP): connections with host autophagy. Cells 1, 396–408. 10.3390/cells1030396 24710482 PMC3901117

[B90] Lam K. K. Zheng X. Forestieri R. Balgi A. D. Nodwell M. Vollett S. (2012). Nitazoxanide stimulates autophagy and inhibits mTORC1 signaling and intracellular proliferation of *Mycobacterium tuberculosis* . PLoS Pathog. 8, e1002691. 10.1371/journal.ppat.1002691 22589723 PMC3349752

[B91] Laws T. R. Clark G. C. D’elia R. V. (2015). Immune profiling of the progression of a Balb/C mouse aerosol infection by burkholderia pseudomallei and the therapeutic implications of targeting HMGB1. Int. J. Infect. Dis. 40, 1–8. 10.1016/j.ijid.2015.09.003 26358857

[B92] Lee C. R. Zeldin D. C. (2015). Resolvin infectious inflammation by targeting the host response. N. Engl. J. Med. 373, 2183–2185. 10.1056/NEJMcibr1511280 26605933 PMC5512422

[B93] Lee Y. S. Han S. H. Lee S. H. Kim Y. G. Park C. B. Kang O. H. (2012). The mechanism of antibacterial activity of tetrandrine against *Staphylococcus aureus* . Foodborne Pathog. Dis. 9 (8), 686–691. 10.1089/fpd.2011.1119 22845553

[B94] Lee M. N. Ye C. Villani A.-C. Raj T. Li W. Eisenhaure T. M. (2014). Common genetic variants modulate pathogen-sensing responses in human dendritic cells. Science 343 (6175), 1246980. 10.1126/science.1246980 24604203 PMC4124741

[B95] Levin-Reisman I. Ronin I. Gefen O. Braniss I. Shoresh N. Balaban N. Q. (2017). Antibiotic tolerance facilitates the evolution of resistance. Science 355, 826–830. 10.1126/science.aaj2191 28183996

[B96] Li N. Xie W. P. Kong H. Min R. Hu C. M. Zhou X. B. (2015). Enrichment of regulatory T-cells in blood of patients with multidrug-resistant tuberculosis. Int. J. Tuberc. Lung Dis. 19, 1230–1238. 10.5588/ijtld.15.0148 26459539

[B97] Li G. He D. Cai X. Guan W. Zhang Y. Wu J. Q. (2023). Advances in the development of phosphodiesterase-4 inhibitors. Eur. J. Med. Chem. 250, 115195. 10.1016/j.ejmech.2023.115195 36809706

[B98] Lin C. C. Wang J. Y. Pu Y. S. (2013). Active tuberculosis during temsirolimus and bevacizumab treatment. J. Clin. Oncol. 31, E18–E20. 10.1200/jco.2012.43.7566 23169507

[B99] Lin A. E. Beasley F. C. Olson J. Keller N. Shalwitz R. A. Hannan T. J. (2015). Role of hypoxia inducible factor-1alpha (HIF-1alpha) in innate defense against uropathogenic *Escherichia coli* infection. PLoS Pathog. 11, e1004818. 10.1371/journal.ppat.1004818 25927232 PMC4415805

[B100] Liu X. Foo G. Lim W. P. Ravikumar S. Sim S. H. Win M. S. (2014). Sulphonylurea usage in melioidosis is associated with severe disease and suppressed immune response. PLoS Negl. Trop. Dis. 8, e2795. 10.1371/journal.pntd.0002795 24762472 PMC3998927

[B101] Liu C. Fan D. Lei Q. Lu A. He X. (2022a). Roles of resolvins in chronic inflammatory response. Int. J. Mol. Sci. 23 (23), 14883. 10.3390/ijms232314883 36499209 PMC9738788

[B102] Liu X. Wu Y. Mao C. Shen J. Zhu K. (2022b). Host-acting antibacterial compounds combat cytosolic bacteria. Trends Microbiol. 30 (8), 761–777. 10.1016/j.tim.2022.01.006 35140036

[B103] Lo J. H. Kulp S. K. Chen C. S. Chiu H. C. (2014). Sensitization of intracellular *Salmonella enterica* serovar typhimurium to aminoglycosides *in vitro* and *in vivo* by a host-targeted antimicrobial agent. Antimicrob. Agents Chemother. 58, 7375–7382. 10.1128/AAC.03778-14 25267669 PMC4249550

[B104] Lv K. Liang Q. (2025). Macrophages in sepsis-induced acute lung injury: exosomal modulation and therapeutic potential. Front. Immunol. 15, 1518008. 10.3389/fimmu.2024.1518008 PMC11746006 39840035

[B105] Maiga M. Ammerman N. C. Maiga M. C. Tounkara A. Siddiqui S. Polis M. (2013). Adjuvant host-directed therapy with types 3 and 5 but not type 4 phosphodiesterase inhibitors shortens the duration of tuberculosis treatment. J. Infect. Dis. 208, 512–519. 10.1093/infdis/jit187 23641020 PMC3699007

[B106] Mandal M. Pires D. Azevedo-Pereira J. M. Anes E. (2025). Host-directed therapies based on protease inhibitors to control *Mycobacterium tuberculosis* and HIV coinfection. Microorganisms 13 (5), 1040. 10.3390/microorganisms13051040 PMC12113826 40431213

[B107] Manes N. P. Nita-Lazar A. (2018). Application of targeted mass spectrometry in Bottom-up proteomics for systems biology research. J. Proteomics 189, 75–90. 10.1016/j.jprot.2018.02.008 29452276 PMC6089676

[B108] Manes N. P. Shulzhenko N. Nuccio A. G. Azeem S. Morgun A. Nita-Lazar A. (2017). Multi-omics comparative analysis reveals multiple layers of host signaling pathway regulation by the gut microbiota. mSystems 2 (5), e00107–e00117. 10.1128/mSystems.00107-17 29085914 PMC5655592

[B109] March C. Regueiro V. Llobet E. Moranta D. Morey P. Garmendia J. (2010). Dissection of host cell signal transduction during *acinetobacter baumannii*-triggered inflammatory response. PLoS ONE5 5 (4), e10033. 10.1371/journal.pone.0010033 20383325 PMC2850920

[B110] Matusiak M. Van Opdenbosch N. Vande Walle L. Sirard J. C. Kanneganti T. D. Lamkanfi M. (2015). Flagellin-induced NLRC4 phosphorylation primes the inflammasome for activation by NAIP5. Proc. Natl. Acad. Sci. U S A. 112, 1541–1546. 10.1073/pnas.1417945112 25605939 PMC4321312

[B111] Mayanja-Kizza H. Jones-Lopez E. Okwera A. Wallis R. S. Ellner J. J. Mugerwa R. D. (2005). Immunoadjuvant prednisolone therapy for HIV-Associated tuberculosis: a phase 2 clinical trial in Uganda. J. Infect. Dis. 191, 856–865. 10.1086/427995 15717259 PMC4515766

[B112] Mayer-Barber K. D. Andrade B. B. Oland S. D. Amaral E. P. Barber D. L. Gonzales J. (2014). Host-directed therapy of tuberculosis based on interleukin.1 and type I interferon crosstalk. Nature 511 (99), 99–103. 10.1038/nature13489 24990750 PMC4809146

[B113] Memiševic´ V. Zavaljevski N. Pieper R. Rajagopala S. V. Kwon K. Townsend K. (2013). Novel Burkholderia mallei virulence factors linked to specific host-pathogen protein interactions. Mol. Cell Proteomics 12, 3036–3051. 10.1074/mcp.M113.029041 23800426 PMC3820922

[B114] Miek L. Jordan P. M. Gunther K. Pace S. Beyer T. Kowalak D. (2022). *Staphylococcus aureus* controls eicosanoid and specialized pro-resolving mediator production *via* lipoteichoic acid. Immunology 166 (1), 47–67. 10.1111/imm.13449 35143048 PMC9426618

[B115] Miller M. Dreisbach A. Otto A. Becher D. Bernhardt J. Hecker M. (2011). Mapping of interactions between human macrophages and Staphylococcus aureus reveals an involvement of MAP kinase signaling in the host defense. J. Proteome Res. 10 (9), 4018–4032. 10.1021/pr200224x 21736355

[B116] Misra U. K. Kalita J. Nair P. P. (2010). Role of aspirin in tuberculous meningitis: a randomized open label placebo controlled trial. J. Neurol. Sci. 293, 12–17. 10.1016/j.jns.2010.03.025 20421121

[B117] Mizushima N. (2007). Autophagy: process and function. Genes Dev. 21, 2861–2873. 10.1101/gad.1599207 18006683

[B118] Moreland L. W. (2004). Drugs that block tumour necrosis factor: experience in patients with rheumatoid arthritis. Pharmacoeconomics 22 (2), 39–53. 10.2165/00019053-200422001-00005 15157003

[B119] Moriel D. G. Piccioli D. Raso M. M. Pizza M. (2024). The overlooked bacterial pandemic. Semin. Immunopathol. 45 (4−6), 481–491. 10.1007/s00281-023-00997-1 PMC11136754 38078911

[B120] Morris F. C. DexterC K. X. UddinMI P. A. Y. (2019). The mechanisms of disease caused by *Acinetobacter baumannii* . Front. Microbiol. 10, 1601. 10.3389/fmicb.2019.01601 PMC6650576 31379771

[B121] Morte-Romea E. Pesini C. Pellejero-Sagastizábal G. Letona-Giménez S. Martínez-Lostao L. Aranda S. L. (2024). CAR immunotherapy for the treatment of infectious diseases: a systematic review. Front. Immunol. 15, 1289303. 10.3389/fimmu.2024.1289303 PMC10861799 38352878

[B122] Napier R. J. Rafi W. Cheruvu M. Powell K. Zaunbrecher M. Bornmann W. (2011). Imatinib-sensitive tyrosine kinases regulate mycobacterial pathogenesis and represent therapeutic targets against. Tuberc. Cell HostMicrobe 10, 475–485. 10.1016/j.chom.2011.09.010 22100163 PMC3222875

[B123] Napier R. J. Norris B. A. Swimm A. Giver C. R. Harris W. A. C. Laval J. (2015). Low doses of imatinib induce myelopoiesis and enhance host anti-microbial immunity. PLoS Pathog. 11, e1004770. 10.1371/journal.ppat.1004770 25822986 PMC4379053

[B124] Nathan C. Shiloh M. U. (2000). Reactive oxygen and nitrogen intermediates in the relationship between Mammalian hosts and microbial pathogens. Proc. Natl. Acad. Sci. U S A. 97, 8841–8848. 10.1073/pnas.97.16.8841 10922044 PMC34021

[B125] Netea M. G. Domínguez-Andrés J. Barreiro L. B. Chavakis T. Divangahi M. Fuchs E. (2020). Defining trained immunity and its role in health and disease. Nat. Rev. Immunol. 20 (6), 375–388. 10.1038/s41577-020-0285-6 32132681 PMC7186935

[B126] Orozco L. D. Bennett B. J. Farber C. R. Ghazalpour A. Pan C. Che N. (2012). Unraveling inflammatory responses using systems genetics and gene-environment interactions in macrophages. Cell 151 (3), 658–670. 10.1016/j.cell.2012.08.043 23101632 PMC3513387

[B127] Ou J. Li K. Yuan H. Du S. Wang T. Deng Q. (2025). *Staphylococcus aureus* vesicles impair cutaneous wound healing through p38 MAPK-MerTK cleavage-mediated inhibition of macrophage efferocytosis. Cell Commun. Signal CCS 23 (1), 14. 10.1186/s12964-024-01994-z PMC11708000 39780180

[B128] Pahuja I. Verma A. Ghoshal A. Mukhopadhyay S. Kumari A. Shaji A. (2023). Biapenem, a carbapenem antibiotic, elicits mycobacteria specific immune responses and reduces the recurrence of tuberculosis. Microbiol. Spectr. 11, e0085823. 10.1128/spectrum.00858-23 37272833 PMC10434282

[B129] Painuli S. Semwal P. Sharma R. Akash S. (2023). Superbugs or multidrug resistant microbes: a new threat to the society. Health Sci. Rep. 6 (8), e1480. 10.1002/hsr2.1480 PMC10397562 37547359

[B130] Palazon A. Goldrath A. W. Nizet V. Johnson R. S. (2014). HIF transcription factors, inflammation, and immunity. Immunity 41 (4), 518–528. 10.1016/j.immuni.2014.09.008 25367569 PMC4346319

[B131] Pan T. Li M. Guo J. Zhao D. Liu X. Huang H. (2025). Bacillus safensis from sauerkraut alleviates acute lung injury induced by methicillin-resistant Staphylococcus aureus through the regulation of M2 macrophage polarization *via* its metabolite esculin. J. Agric. Food Chem. 73 (1), 409–424. 10.1021/acs.jafc.4c05508 39723899

[B132] Papalexi E. Satija R. (2018). Single-cell RNA sequencing to explore immune cell heterogeneity. Nat. Rev. Immunol. 18, 35–45. 10.1038/nri.2017.76 28787399

[B133] Parihar S. P. Guler R. Khutlang R. Lang D. M. Hurdayal R. Mhlanga M. M. (2014). Statin therapy reduces the *Mycobacterium tuberculosis* burden in human macrophages and in mice by enhancing autophagy and phagosome maturation. J. Infect. Dis. 209, 754–763. 10.1093/infdis/jit550 24133190

[B134] Parihar S. P. Guler R. Brombacher F. (2019). Statins: a viable candidate for host-directed therapy against infectious diseases. Nat. Rev. Immunol. 19, 104–117. 10.1038/s41577-018-0094-3 30487528

[B135] Park D. W. Jiang S. Tadie J.-M. Stigler W. S. Gao Y. Deshane J. (2013). Activation of AMP-activated protein kinase enhances neutrophil chemotaxis and bacterial killing. Mol. Med. 19 (1), 387–398. 10.2119/molmed.2013.00065 24091934 PMC3883969

[B136] Park S. H. Kang K. Giannopoulou E. Qiao Y. Kang K. Kim G. (2017). Type I interferons and the cytokine TNF cooperatively reprogram the macrophage epigenome to promote inflammatory activation. Nat. Immunol. 18 (10), 1104–1116. 10.1038/ni.3818 28825701 PMC5605457

[B137] Parra-Millan R. al A.-A. R. (2018). Intracellular trafficking and persistence of *Acinetobacter baumannii* requires transcription factor EB. mSphere3 (2), e00106–e00118. 10.1128/mSphere.00106-18 PMC5874439 29600279

[B138] Paton N. I. Gurumurthy M. Lu Q. Leek F. Kwan P. Koh H. W. L. (2024). Adjunctive pascolizumab in rifampicin-susceptible pulmonary tuberculosis: proof-Of-concept, partially-randomized, double-blind, placebo-controlled, dose-escalation trial. J. Infect. Dis. 230 (3), 590–597. 10.1093/infdis/jiae104 38527849

[B139] Pello O. M. (2016). Macrophages and c-Myc cross paths. Oncoimmunology 5, e1151991. 10.1080/2162402X.2016.1151991 27471623 PMC4938353

[B140] Pello O. M. De Pizzol M. Mirolo M. Soucek L. Zammataro L. Amabile A. (2012). Role of c-MYC in alternative activation of human macrophages and tumor-associated macrophage biology. Blood 119, 411–421. 10.1182/blood-2011-02-339911 22067385

[B141] Peng K. T. Hsieh C. C. Huang T. Y. Chen P. C. Shih H. N. Lee M. S. (2017). *Staphylococcus aureus* biofilm elicits the expansion, activation and polarization of myeloid-derived suppressor cells *in vivo* and *in vitro* . PLoS One 12 (8), e0183271. 10.1371/journal.pone.0183271 28813499 PMC5559065

[B142] Peyssonnaux C. Datta V. Cramer T. Doedens A. Theodorakis E. A. Gallo R. L. (2005). HIF-1alpha expression regulates the bactericidal capacity of phagocytes. J. Clin. Invest. 115, 1806–1815. 10.1172/JCI23865 16007254 PMC1159132

[B143] Pielage J. F. Powell K. R. Kalman D. Engel J. N. (2008). RNAi screen reveals an abl kinase-dependent host cell pathway involved in *Pseudomonas aeruginosa* internalization. PLoS Pathog. 4, e1000031. 10.1371/journal.ppat.1000031 18369477 PMC2265438

[B144] Pires D. Valente S. Calado M. Mandal M. Azevedo-Pereira J. M. Anes E. (2021). Repurposing saquinavir for host-directed therapy to control mycobacterium tuberculosis infection (in eng). Front. Immunol. 12, 647728. 10.3389/fimmu.2021.647728 PMC8032898 33841429

[B145] Pizzolla A. Hultqvist M. Nilson B. Grimm M. J. Eneljung T. Jonsson I. M. (2012). Reactive oxygen species produced by the NADPH oxidase 2 complex in monocytes protect mice from bacterial infections. J. Immunol. 188, 5003–5011. 10.4049/jimmunol.1103430 22491245 PMC3566649

[B146] Poerio N. Olimpieri T. Henrici De Angelis L. De Santis F. Thaller M. C. D'Andrea M. M. (2022). Fighting MDR-*Klebsiella pneumoniae* infections by a combined Host- and pathogen-directed therapeutic approach. Front. Immunol. 13, 835417. 10.3389/fimmu.2022.835417 PMC8884248 35237274

[B147] Pollock J. D. Williams D. A. Gifford M. A. Li L. L. Du X. Fisherman J. (1995). Mouse model of X-linked chronic granulomatous disease, an inherited defect in phagocyte superoxide production. Nat. Genet. 9 (2), 202–209. 10.1038/ng0295-202 7719350

[B148] Preston J. A. Bewley M. A. Marriott H. M. McGarry Houghton A. Mohasin M. Jubrail J. (2019). Alveolar macrophage apoptosis-associated bacterial killing helps prevent murine pneumonia. Am. J. Respir. Crit. Care Med. 200, 84–97. 10.1164/rccm.201804-0646OC 30649895 PMC6603058

[B149] Qi X. Man S. M. Malireddi R. K. Karki R. Lupfer C. Gurung P. (2016). Cathepsin B modulates lysosomal biogenesis and host defense against Francisella novicida infection. J. Exp. Med. 213, 2081–2097. 10.1084/jem.20151938 27551156 PMC5030800

[B150] Qin Q. M. Pei J. Ancona V. Shaw B. D. Ficht T. A. de Figueiredo P. (2008). RNAi screen of endoplasmic reticulum-associated host factors reveals a role for IRE1alpha in supporting Brucella replication. PLoS Pathog. 4, e1000110. 10.1371/journal.ppat.1000110 18654626 PMC2453327

[B151] Rabadi S. M. Sanchez B. C. Varanat M. Ma Z. Catlett S. V. Melendez J. A. (2016). Antioxidant defenses of Francisella tularensis modulate macrophage function and production of proinflammatory cytokines. J. Biol. Chem. 291, 5009–5021. 10.1074/jbc.M115.681478 26644475 PMC4777838

[B152] Rajabalee N. Siushansian H. Weerapura M. Berton S. Berbatovci F. Hooks B. (2023). ATF2 orchestrates macrophage differentiation and activation to promote antibacterial responses. J. Leukoc. Biol. 114, 280–298. 10.1093/jleuko/qiad076 37403209

[B153] Rinchai D. Riyapa D. Buddhisa S. Utispan K. Titball R. W. Stevens M. P. (2015). Macroautophagy is essential for killing of intracellular Burkholderia pseudomallei in human neutrophils. Autophagy 11, 748–755. 10.1080/15548627.2015.1040969 25996656 PMC4509438

[B154] Rius J. Guma M. Schachtrup C. Akassoglou K. Zinkernagel A. S. Nizet V. (2008). NF-kappaB links innate immunity to the hypoxic response through transcriptional regulation of HIF-1alpha. Nature 453, 807–811. 10.1038/nature06905 18432192 PMC2669289

[B155] Robles-Medranda C. (2016). Confocal endomicroscopy: is it time to move on? World J. Gastrointest. Endosc. 8, 1–3. 10.4253/wjge.v8.i1.1 26788257 PMC4707318

[B156] Roca F. J. Ramakrishnan L. (2013). TNF dually mediates resistance and susceptibility to mycobacteria *via* mitochondrial reactive oxygen species. Cell 153, 521–534. 10.1016/j.cell.2013.03.022 23582643 PMC3790588

[B157] Ruiz A. Sarabia C. Torres M. Juárez E. (2019). Resolvin D1 (RvD1) and maresin 1 (Mar1) contribute to human macrophage control of M. tuberculosis infection while resolving inflammation. Int. Immunopharmacol. 74, 105694. 10.1016/j.intimp.2019.105694 31226532

[B158] Ryter S. W. Cloonan S. M. Choi A. M. (2013). Autophagy: a critical regulator of cellular metabolism and homeostasis. Mol. Cells 36, 7–16. 10.1007/s10059-013-0140-8 23708729 PMC3887921

[B159] Sadikot R. T. Zeng H. Yull F. E. Li B. Cheng D. S. Kernodle D. S. (2004). p47(phox) deficiency impairs NF-kappaB activation and host defense in Pseudomonas pneumonia. J. Immunol. 172, 1801–1808. 10.4049/jimmunol.172.3.1801 14734763

[B160] Saharia K. K. Petrovas C. Ferrando-Martinez S. Leal M. Luque R. Ive P. (2016). Tuberculosis therapy modifies the cytokine profile, maturation state, and expression of inhibitory molecules on mycobacterium tuberculosis-specific CD4+ T-Cells. PloS One 11, e0158262. 10.1371/journal.pone.0158262 27367521 PMC4930205

[B161] Schaffer K. Taylor C. T. (2015). The impact of hypoxia on bacterial infection. FEBS J. 282, 2260–2266. 10.1111/febs.13270 25786849

[B162] Schieber M. Chandel N. S. (2014). ROS function in redox signaling and oxidative stress. Curr. Biol. 24 (10), R453–R462. 10.1016/j.cub.2014.03.034 24845678 PMC4055301

[B163] Schnaith A. Kashkar H. Leggio S. A. Addicks K. Krönke M. Krut O. (2007). *Staphylococcus aureus* subvert autophagy for induction of caspase-independent host cell death. J. Biol. Chem. 282 (4), 2695–2706. 10.1074/jbc.M609784200 17135247

[B164] Schoeman J. F. Springer P. van Rensburg A. J. Swanevelder S. Hanekom W. A. Haslett P. A. J. (2004). Adjunctive thalidomide therapy for childhood tuberculous meningitis: results of a randomized study. J. Child. Neurol. 19 (250), 257. 10.1177/088307380401900402 15163089

[B165] Schoeman J. F. van Rensburg A. J. Laubscher J. A. Springer P. (2011). The role of aspirin in childhood tuberculous meningitis. J. Child. Neurol. 26, 956–962. 10.1177/0883073811398132 21628697

[B166] Schwegmann A. Brombacher F. (2008). Host-directed drug targeting of factors hijacked by pathogens. Sci. Signal 1, re81. 10.1126/scisignal.129re8 18648074

[B167] Schweppe D. K. Harding C. Chavez J. D. Wu X. Ramage E. Singh P. K. (2015). Host-microbe protein interactions during bacterial infection. Chem. Biol. 22, 1521–1530. 10.1016/j.chembiol.2015.09.015 26548613 PMC4756654

[B168] Scott C. C. Cuellar-Mata P. Matsuo T. Davidson H. W. Grinstein S. (2002). Role of 3-phosphoinositides in the maturation of Salmonella-containing vacuoles within host cells. J. Biol. Chem. 277 (15), 12770–12776. 10.1074/jbc.M110399200 11821391

[B169] Shapira T. Christofferson M. Av-Gay Y. (2024). The antimicrobial activity of innate host-directed therapies: a systematic review. Int. J. Antimicrob. Agents 63 (5), 107138. 10.1016/j.ijantimicag.2024.107138 38490573

[B170] Shi L. Jiang Q. Bushkin Y. Subbian S. Tyagi S. (2019). Biphasic dynamics of macrophage immunometabolism during *Mycobacterium tuberculosis* infection. mBio 10, e02550-18. 10.1128/mBio.02550-18 30914513 PMC6437057

[B171] Silva O. N. de la Fuente-Núñez C. Haney E. F. Fensterseifer I. C. Ribeiro S. M. Porto W. F. (2016). An anti-infective synthetic peptide with dual antimicrobial and immunomodulatory activities. Sci. Rep. 6, 35465. 10.1038/srep35465 PMC5090204 27804992

[B172] Singh V. K. Khan A. Xu Y. Mai S. Zhang L. Mishra A. (2022a). Antibody-mediated LILRB2-receptor antagonism induces human myeloid-derived suppressor cells to kill *Mycobacterium tuberculosis* . Front. Immunol. 13, 865503. 10.3389/fimmu.2022.865503 PMC9229593 35757769

[B173] Singh M. Kumar S. Singh B. Jain P. Kumari A. Pahuja I. (2022b). The 1, 2-ethylenediamine SQ109 protects against tuberculosis by promoting M1 macrophage polarization through the p38 MAPK pathway. Commun. Biol. 5, 2. 10.1038/s42003-022-03693-2 PMC9334294 35902694

[B174] Singhal A. Jie L. Kumar P. Hong G. S. Leow M. K. Paleja B. (2014). Metformin as adjunct antituberculosis therapy. Sci. Transl. Med. 6 (263), 263ra159. 10.1126/scitranslmed.3009885 25411472

[B175] Singhania A. Wilkinson R. J. Rodrigue M. Haldar P. O'Garra A. (2018). The value of transcriptomics in advancing knowledge of the immune response and diagnosis in tuberculosis. Nat. Immunol. 19 (11), 1159–1168. 10.1038/s41590-018-0225-9 30333612 PMC6554194

[B176] Singothu S. Kinkar D. Vulli D. Brahma U. Bandaru R. K. Bhandari V. (2025). Dysregulation of host histone acetylation by *acinetobacter baumannii*: a novel mechanism for autophagy inhibition and bacterial survival. ACS Infect. Dis. 11 (7), 1922–1933. 10.1021/acsinfecdis.5c00080 40509567

[B177] Sjoelund V. Smelkinson M. Nita-Lazar A. (2014). Phosphoproteome profiling of the macrophage response to different toll-like receptor ligands identifies differences in global phosphorylation dynamics. J. Proteome Res. 13 (11), 5185–5197. 10.1021/pr5002466 24941444 PMC4227906

[B178] Snelgrove R. J. Goulding J. Didierlaurent A. M. Lyonga D. Vekaria S. Edwards L. (2008). A critical function for cd200 in lung immune homeostasis and the severity of influenza infection. Nat. Immunol. 9, 1074–1083. 10.1038/ni.1637 18660812

[B179] Sogi K. M. Lien K. A. Johnson J. R. Krogan N. J. Stanley S. A. (2017). The tyrosine kinase inhibitor gefitinib restricts *Mycobacterium tuberculosis* growth through increased lysosomal biogenesis and modulation of cytokine signaling. ACS Infect. Dis. 3 (8), 564–574. 10.1021/acsinfecdis.7b00046 28537707 PMC5728371

[B180] Stanley S. A. Barczak A. K. Silvis M. R. Luo S. S. Sogi K. Vokes M. (2014). Identification of host-targeted small molecules that restrict intracellular *Mycobacterium tuberculosis* growth. PLoS Pathog. 10 (2), e1003946. 10.1371/journal.ppat.1003946 24586159 PMC3930586

[B181] Stubbington M. J. T. Rozenblatt-Rosen O. Regev A. Teichmann S. A. (2017). Single-cell transcriptomics to explore the immune system in health and disease. Science 358, 58–63. 10.1126/science.aan6828 28983043 PMC5654495

[B182] Subbarao S. Sanchez-Garrido J. Krishnan N. Shenoy A. R. Robertson B. D. (2020). Genetic and pharmacological inhibition of inflammasomes reduces the survival of *Mycobacterium tuberculosis* strains in macrophages. Sci. Rep. 10 (1), 3709. 10.1038/s41598-020-60560-y 32111888 PMC7048741

[B183] Subbian S. Tsenova L. O'Brien P. Yang G. Koo M. S. Peixoto B. (2011a). Phosphodiesterase-4 inhibition combined with isoniazid treatment of rabbits with pulmonary tuberculosis reduces macrophage activation and lung pathology. Am. J. Pathol. 179, 289–301. 10.1016/j.ajpath.2011.03.039 PMC3123788 21703411

[B184] Subbian S. Tsenova L. O'Brien P. Yang G. Koo M. S. Peixoto B. (2011b). Phosphodiesterase-4 inhibition alters gene expression and improves isoniazid – mediated clearance of *Mycobacterium tuberculosis* in rabbit lungs. PLoS Pathog. 7, e1002262. 10.1371/journal.ppat.1002262 PMC3174258 21949656

[B185] Subbian S. Koo M. S. Tsenova L. Khetani V. Zeldis J. B. Fallows D. (2016a). Pharmacologic inhibition of host phosphodiesterase.4 improves isoniazid-mediated clearance of *Mycobacterium tuberculosis* . Front. Immunol. 7, 238. 10.3389/fimmu.2016.00238 27379099 PMC4911353

[B186] Subbian S. Tsenova L. Holloway J. Peixoto B. O'Brien P. Dartois V. (2016b). Adjunctive phosphodiesterase.4 inhibitor therapy improves antibiotic response to pulmonary tuberculosis in a rabbit model. EBioMedicine 4 (104), 114. 10.1016/j.ebiom.2016.01.015 26981575 PMC4776074

[B187] Sutera V. Levert M. Burmeister W. P. Schneider D. Maurin M. (2014). Evolution toward high-level fluoroquinolone resistance in francisella species. J. Antimicrob. Chemother. 69, 101–110. 10.1093/jac/dkt321 23963236

[B188] Takeuchi O. Akira S. (2010). Pattern recognition receptors and inflammation. Cell 140, 805–820. 10.1016/j.cell.2010.01.022 20303872

[B189] Tasneen R. Mortensen D. S. Converse P. J. Urbanowski M. E. Upton A. Fotouhi N. (2021). Dual mTORC1/mTORC2 inhibition as a host-directed therapeutic target in pathologically distinct mouse models of tuberculosis. Antimicrob. Agents Chemother. 65, e0025321. 10.1128/AAC.00253-21 PMC8373221 33903099

[B190] Taylor C. T. Colgan S. P. (2017). Regulation of immunity and inflammation by hypoxia in immunological niches. Nat. Rev. Immunol. 17, 774–785. 10.1038/nri.2017.103 28972206 PMC5799081

[B191] Teo K. Y. W. Zhang S. Loh J. T. Lai R. C. Hey H. W. D. Lam K. P. (2023). Mesenchymal stromal cell exosomes mediate M2-like macrophage polarization through CD73/ecto-5’-nucleotidase activity. Pharmaceutics 15 (5), 1489. 10.3390/pharmaceutics15051489 PMC10220822 37242732

[B192] Thom R. E. Williamson E. D. Casulli J. Butcher W. A. Burgess G. Laws T. R. (2023). Assessment of cd200r activation in combination with doxycycline in A model of melioidosis. Microbiol. Spectr. 11, E0401622. 10.1128/spectrum.04016-22 37199641 PMC10269878

[B193] Thomas-Rüddel D. Giamarellos-Bourboulis E. Neumann C. Briegel J. Roquilly A. Annane D. (2025). IFNγ in human sepsis: a scoping review. Ann. Intensive Care 15 (1), 112. 10.1186/s13613-025-01534-z PMC12325153 40762872

[B194] Thurlow L. R. Hanke M. L. Fritz T. Angle A. Aldrich A. Williams S. H. (2011). *Staphylococcus aureus* biofilms prevent macrophage phagocytosis and attenuate inflammation *in vivo* . J. Immunol. Balt. 186 (11), 6585–6596. 10.4049/jimmunol.1002794 21525381 PMC3110737

[B195] Tian N. Chu H. Li Q. Sun H. Zhang J. Chu N. (2025). Host-directed therapy for tuberculosis. Eur. J. Med. Res. 30 (1), 267. 10.1186/s40001-025-02443-4 PMC11987284 40211397

[B196] Tjelle T. E. Brech A. Juvet L. K. Griffiths G. Berg T. (1996). Isolation and characterization of early endosomes, late endosomes and terminal lysosomes: their role in protein degradation. J. Cell Sci. 109, 2905–2914. 10.1242/jcs.109.12.2905 9013338

[B197] Tobin D. M. (2015). Host-directed therapies for tuberculosis. Cold Spring Harb. Perspect. Med. 5, a021196. 10.1101/cshperspect.a021196 25986592 PMC4588138

[B198] Tobin D. M. Roca F. J. Oh S. F. McFarland R. Vickery T. W. Ray J. P. (2012). Host genotype-specific therapies can optimize the inflammatory response to mycobacterial infections. Cell 148, 434–446. 10.1016/j.cell.2011.12.023 22304914 PMC3433720

[B199] Tramontana J. M. Utaipat U. Molloy A. Akarasewi P. Burroughs M. Makonkawkeyoon S. (1995). Thalidomide treatment reduces tumor necrosis factor-α production and enhances weight gain in patients with pulmonary tuberculosis. Mol. Med. 1, 384.397–397. 10.1007/bf03401576 PMC2229989 8521296

[B200] Turner J. Frank A. A. Brooks J. V. Marietta P. M. Orme I. M. (2001). Pentoxifylline treatment of mice with chronic pulmonary tuberculosis accelerates the development of destructive pathology. Immunology 102, 248–253. 10.1046/j.1365-2567.2001.01161.x 11260331 PMC1783172

[B201] Van Acker H. Coenye T. (2017). The role of reactive oxygen species in antibiotic-mediated killing of bacteria. Trends Microbiol. 25 (6), 456–466. 10.1016/j.tim.2016.12.008 28089288

[B202] van den Biggelaar RHGA Walburg K. V. van den Eeden S. J. F. van Doorn C. L. R. Meiler E. de Ries A. S. (2024a). Identification of kinase inhibitors as potential host-directed therapies for intracellular bacteria. Sci. Rep. 14 (1), 17225. 10.1038/s41598-024-68102-6 PMC11282061 39060313

[B203] van den Biggelaar R. Walburg K. V. van den Eeden S. J. F. van Doorn C. L. R. Meiler E. de Ries A. S. (2024b). Identification of kinase modulators as host-directed therapeutics against intracellular Methicillin-resistant *Staphylococcus aureus* infection. Front. Cell Infect. Microbiol. 14, 1367938. 10.3389/fcimb.2024.1367938 38590439 PMC10999543

[B204] van Doorn C. L. R. Steenbergen S. A. M. Walburg K. V. Ottenhoff T. H. M. (2021). Pharmacological poly (ADP-Ribose) polymerase inhibitors decrease *Mycobacterium tuberculosis* survival in human macrophages. Front. Immunol. 12, 712021. 10.3389/fimmu.2021.712021 PMC8662539 34899683

[B205] Vargas J. G. Wagner J. Shaikh H. Lang I. Medler J. Anany M. (2022). A TNFR2-specific TNF fusion protein with improved *in vivo* activity. Front. Immunol. 13, 888274. 10.3389/fimmu.2022.888274 PMC9234581 35769484

[B206] Vazquez-Torres A. Xu Y. Jones-Carson J. Holden D. W. Lucia S. M. Dinauer M. C. (2000). Salmonella pathogenicity island 2-dependent evasion of the phagocyte NADPH oxidase. Science 287, 1655–1658. 10.1126/science.287.5458.1655 10698741

[B207] Venkatesan K. Rual J. F. Vazquez A. Stelzl U. Lemmens I. Hirozane-Kishikawa T. (2009). An empirical framework for binary interactome mapping. Nat. Methods 6 (1), 83–90. 10.1038/nmeth.1280 19060904 PMC2872561

[B208] Verma R. N. Zubbair Malik Md. Singh G. P. Subbarao N. (2022). Identification of key proteins in host–pathogen interactions between *Mycobacterium tuberculosis* and homo sapiens: a systematic network theoretical approach. Healthc. Anal. 2, 100052. 10.1016/j.health.2022.100052

[B209] Vozza E. G. Mulcahy M. E. McLoughlin R. M. (2021). Making the Most of the host: targeting the autophagy pathway facilitates *Staphylococcus aureus* infection intracellular survival in neutrophils. Front. Immunol. 12, 667387. 10.3389/fimmu.2021.667387 34220813 PMC8242348

[B210] Walker N. F. Clark S. O. Oni T. Andreu N. Tezera L. Singh S. (2012). Doxycycline and HIV infection suppress tuberculosis-induced matrix metalloproteinases. Am. J. Respir. Crit. Care 185, 989–997. 10.1164/rccm.201110-1769oc 22345579 PMC3359940

[B211] Wallis R. S. Hafner R. (2015). Advancing host-directed therapy for tuberculosis. Nat. Rev. Immunol. 15, 255–263. 10.1038/nri3813 25765201

[B212] Wallis R. S. Nsubuga P. Whalen C. Mugerwa R. D. Okwera A. Oette D. (1996). Pentoxifylline therapy in human immunodeficiency virus-seropositive persons with tuberculosis: a randomized, controlled trial. J. Infect. Dis. 174, 727–733. 10.1093/infdis/174.4.727 8843209

[B213] Wallis R. S. Kyambadde P. Johnson J. L. Horter L. Kittle R. Pohle M. (2004). A study of the safety, immunology, virology, and microbiology of adjunctive etanercept in HIV.1. Associated Tuberculosis. Aids 18, 257.264. 10.1097/00002030-200401230-00015 15075543

[B214] Wallis R. S. van Vuuren C. Potgieter S. (2009). Adalimumab treatment of life-threatening tuberculosis. Clin. Infect. Dis. 48, 1429–1432. 10.1086/598504 19364287

[B215] Wallis R. S. Maeurer M. Mwaba P. Chakaya J. Rustomjee R. Migliori G. B. (2016). Tuberculosis-advances in development of new drugs, treatment regimens, host directed therapies, and biomarkers. Lancet Infect. Dis. 16, E34–E46. 10.1016/S1473-3099(16)00070-0 27036358

[B216] Wallis R. S. Ginindza S. Beattie T. Arjun N. Likoti M. Edward V. A. (2021a). Adjunctive host-directed therapies for pulmonary tuberculosis: a prospective, open-label, phase 2, randomised controlled trial. Lancet Respir. Med. 9 (8), 897–908. 10.1016/S2213-2600(20)30448-3 PMC8332197 33740465

[B217] Wallis R. S. Ginindza S. Beattie T. Arjun N. Likoti M. Edward V. A. (2021b). Adjunctive host-directed therapies for pulmonary tuberculosis: a prospective, openlabel, phase 2, randomised controlled trial. Lancet Respir. Med. 9, 897–908. 10.1016/s2213-2600(20)30448-3 33740465 PMC8332197

[B218] Wang Y. Sun Q. Zhang Y. Li X. Liang Q. Guo R. (2023). Systemic immune dysregulation in severe tuberculosis patients revealed by a single-cell transcriptome atlas. J. Infect. 86 (5), 421–438. 10.1016/j.jinf.2023.03.020 37003521

[B219] Webber J. L. Tooze S. A. (2010). Coordinated regulation of autophagy by p38alpha MAPK through mAtg9 and p38IP. EMBO J. 29, 27–40. 10.1038/emboj.2009.321 19893488 PMC2808369

[B220] Weintz G. Olsen J. V. Frühauf K. Niedzielska M. Amit I. Jantsch J. (2010). The phosphoproteome of toll-like receptor-activated macrophages. Mol. Syst. Biol. 6, 371. 10.1038/msb.2010.29 20531401 PMC2913394

[B221] Windels E. M. Michiels J. E. Fauvart M. Wenseleers T. Van den Bergh B. (2019). Bacterial persistence promotes the evolution of antibiotic resistance by increasing survival and mutation rates. Isme J. 13, 1239–1251. 10.1038/s41396-019-0344-9 30647458 PMC6474225

[B222] Wong D. NielsenTB B. R. A. PantapalangkoorP L. B. (2017). SpellbergB. Clinical and pathophysiological overview of *acinetobacter* infections: a century of challenges. Clin. Microbiol. Rev. 30 (1), 409–447. 10.1128/CMR.00058-16 PMC5217799 27974412

[B223] Xiong Q. Lin M. Rikihisa Y. (2009). Cholesterol-dependent anaplasma phagocytophilum exploits the low-density lipoprotein uptake pathway. PLoS Pathog. 5 (3), e1000329. 10.1371/journal.ppat.1000329 19283084 PMC2654415

[B224] Xue Y. Yun D. Esmon A. Zou P. Zuo S. Yu Y. (2008). Proteomic dissection of Agonist- specific TLR-mediated inflammatory responses on macrophages at subcellular resolution. J. Proteome Res. 7 (8), 3180–3193. 10.1021/pr800021a 18572962

[B225] Yang H. Ke Y. Wang J. Tan Y. Myeni S. K. Li D. (2011). Insight into bacterial virulence mechanisms against host immune response *via* the yersinia pestishuman protein-protein interaction network. Infect. Immun. 79, 4413–4424. 10.1128/IAI.05622-11 21911467 PMC3257920

[B226] Youn C. Pontaza C. Wang Y. Dikeman D. A. Joyce D. P. Alphonse M. P. (2023). Neutrophil-intrinsic tumor necrosis factor receptor signaling orchestrates host defense against *Staphylococcus aureus* infection. Sci. Adv. 9 (24), eadf8748. 10.1126/sciadv.adf8748 PMC10275602 37327341

[B227] Yu J. Yuan H. Guo J. Dong Z. Li S. Fu Q. (2023a). Combining multi-omics analysis to identify host-targeted targets for the control of brucella infection. Microb. Biotechnol. 16 (12), 2345–2366. 10.1111/1751-7915.14307 PMC10686141 37882474

[B228] Yu F. Y. Zheng K. Wu Y. F. Gao S. W. Weng Q. Y. Zhu C. (2023b). Rapamycin exacerbates *Staphylococcus aureus* pneumonia by inhibiting mTOR-RPS6 in macrophages. J. Inflamm. Res. 16, 5715–5728. 10.2147/JIR.S434483 38053607 PMC10695130

[B229] Zhang L. Li J. Zong L. Chen X. Chen K. Jiang Z. (2016). Reactive oxygen species and targeted therapy for pancreatic cancer. Oxid. Med. Cell Longev. 2016, 1616781. 10.1155/2016/1616781 26881012 PMC4735911

[B230] Zhang Y. Zhu L. Li X. Ge C. Pei W. Zhang M. (2024). M2 macrophage exosome-derived lncRNA AK083884 protects mice from CVB3-induced viral myocarditis through regulating PKM2/HIF-1ƒ¿ axis mediated metabolic reprogramming of macrophages. Redox Biol. 69, 103016. 10.1016/j.redox.2023.103016 PMC10792748 38160539

[B231] Zheng Y. Feng J. Qu Q. Liu Y. Zheng Y. Li Y. (2026). Emerging host-directed strategies for overcoming drug resistance and immune evasion in *Staphylococcus aureus* infections. J. Adv. Res. 83, 1083–1104. 10.1016/j.jare.2025.08.001 40769377 PMC13131439

[B232] Zhu J. Huang J. W. Tseng P. H. Yang Y. T. Fowble J. Shiau C. W. (2004). From the cyclooxygenase-2 inhibitor celecoxib to a novel class of 3-phosphoinositide-dependent protein kinase-1 inhibitors. Cancer Res. 64, 4309–4318. 10.1158/0008-5472.CAN-03-4063 15205346

[B233] Zumla A. Rao M. Wallis R. S. Kaufmann S. H. Rustomjee R. Mwaba P. (2016). Host-directed therapies for infectious diseases: current status, recent progress, and future prospects. Lancet Infect. Dis. 16, e47–e63. 10.1016/s1473-3099(16)00078-5 PMC7164794 27036359

[B234] Zwack E. E. Chen Z. Devlin J. C. Li Z. Zheng X. Weinstock A. (2022). *Staphylococcus aureus* induces a muted host response in human blood that blunts the recruitment of neutrophils. Proc. Natl. Acad. Sci. U S A. 119 (31), e2123017119. 10.1073/pnas.2123017119 PMC9351360 35881802

